# Abnormal Platelet Counts and Clonal Hematopoiesis in the General Population

**DOI:** 10.1097/HS9.0000000000000821

**Published:** 2023-01-05

**Authors:** Priscilla Kamphuis, Maaike G.J.M. van Bergen, Isabelle A. van Zeventer, Aniek O. de Graaf, Avinash G. Dinmohamed, Jonas B. Salzbrunn, Jan Jacob Schuringa, Bert A. van der Reijden, Gerwin Huls, Joop H. Jansen

**Affiliations:** 1 Department of Hematology, University of Groningen, University Medical Center Groningen, the Netherlands; 2 Department of Laboratory Medicine, Laboratory of Hematology, Radboud University Medical Center, Nijmegen, the Netherlands; 3 Department of Research and Development, Netherlands Comprehensive Cancer Organization (IKNL), Utrecht, the Netherlands; 4 Erasmus MC, Department of Public Health, University Medical Center Rotterdam, the Netherlands

## Abstract

Clonal hematopoiesis (CH) is defined by the presence of somatic mutations that may cause clonal expansion of hematopoietic cells. Here, we investigated the association between platelet count abnormalities, CH and consequences on overall survival and the development of hematological malignancies. Individuals with thrombocytopenia (n = 631) or thrombocytosis (n = 178) ≥60 years, and their age- and sex-matched controls, were selected within the population-based Lifelines cohort (n = 167,729). Although the prevalence of CH was not increased in thrombocytopenia cases compared with their controls (37.9% vs 39.3%; *P* = 0.639), mutations in spliceosome genes (*SF3B1*, *SRSF2*, *U2AF1*) were significantly enriched in thrombocytopenia cases (*P* = 0.007). Overall, CH in combination with thrombocytopenia did not impact on survival, but thrombocytopenia in combination with multiple mutated genes (hazard ratio [HR] = 2.08, 95% confidence interval [CI], 1.24-3.50; *P* = 0.006), mutations in *TP53* (HR = 5.83, 95% CI, 2.49-13.64; *P* < 0.001) or spliceosome genes (HR = 2.69, 95% CI, 1.29-5.63; *P* = 0.009) increased the risk of death. The prevalence of CH in thrombocytosis cases was higher compared with controls (55.8% vs 37.7%; *P* < 0.001). Especially mutations in *JAK2* (*P* < 0.001) and *CALR* (*P* = 0.003) were enriched in individuals with thrombocytosis. The presence of CH in individuals with thrombocytosis did not impact on overall survival. However, during follow-up of 11 years 23% of the individuals with thrombocytosis and CH were diagnosed with hematological malignancies. From these, 81% were diagnosed with myeloproliferative disease and 76% carried driver mutations *JAK2*, *CALR*, or *MPL*.

## INTRODUCTION

Throughout life hematopoietic stem and progenitor cells accumulate somatic mutations.^1,2^ When these provide a growth advantage, expanded clones of mutated cells may arise in a process which is called clonal hematopoiesis (CH).^3–5^ CH can frequently be identified in the peripheral blood of older individuals, and is associated with the development of blood cancer, cardiovascular diseases, and all-cause mortality.^3,6–8^

Peripheral blood cytopenias are frequently encountered in the elderly population, and even mild cytopenias are associated with increased morbidity and all-cause mortality.^9,10^ The cause of cytopenias at an older age is multifactorial and cytopenias of different lineages may co-occur. The prevalence of thrombocytopenia (platelet counts <150 × 10^9^/L) increases with aging, and is often an incidental finding.^9,11,12^ It is frequently observed in patients with myelodysplastic syndromes (MDS, 40%–65% of patients).^13–15^ Other causes include a decreased production, autoimmune-related destruction of platelets, and splenic sequestration.^12^ Thrombocytosis (platelet counts >400 × 10^9^/L)^16,17^ is also often an incidental finding and can be caused by a reactive process (called secondary thrombocytosis), familial thrombocytosis, or clonal thrombocytosis.^18^ Clonal thrombocytosis is always observed in cases with essential thrombocythemia (ET) and frequently present in patients with polycythemia vera, both subtypes of myeloproliferative neoplasms (MPNs).^19^ MPNs are a group of clonal diseases that are characterized by an overproduction of terminally differentiated cells of the myeloid lineage and frequently have driver mutations in *JAK2*, *CALR*, or *MPL*.^20^

In combination with CH, cytopenia (as such called clonal cytopenia of undetermined significance [CCUS]) may indicate a bone marrow abnormality and is associated with an increased risk of evolving into a hematological malignancy.^10,21^ Recently, the association between CH and anemia as well as neutropenia and monocytosis has been studied.^7,22,23^ In a population-based case–control study, a higher prevalence of CH, particularly with *SF3B1* and *TP53* mutations, has been observed in older individuals with anemia. The presence of CH did not affect the risk of death in anemic individuals.^7^ Furthermore, it has been shown that the presence of CH in patients with chronic idiopathic neutropenia did not significantly correlate with the severity of neutropenia but mutations in *SRSF2* and *IDH1* were associated with malignant transformation.^22^ Recently, it was shown in a clinical cohort of cases with primary immune thrombocytopenia (ITP, n = 14) that the presence of CH in ITP patients was closely related to disease severity and treatment responsiveness. Previously, it was described that the presence of mutations in *U2AF1* and *PPM1D* are associated with decreased platelet counts, whereas mutations in *DNMT3A*, *CALR*, and *JAK2* are associated with increased platelet counts.^24–26^ However, within the general population, the clinical relevance of CH in individuals with thrombocytopenia or thrombocytosis remains unclear.

By using a case–control study design, we investigated the prevalence of CH and specific mutational patterns within the prospective and population-based Lifelines cohort in individuals (≥60 years) with thrombocytopenia (n = 631) or thrombocytosis (n = 178) and their matched controls. In addition, we studied the impact of CH and specific mutations in the context of abnormal platelet counts on survival and development of hematological malignancies during a follow-up of 11 years.

## MATERIALS AND METHODS

### Study population

This study was performed within the Lifelines Cohort study, which is a multidisciplinary prospective population-based cohort study examining in a unique three-generation design the health and health-related behaviors of 167,729 persons living in the North of the Netherlands. It employs a broad range of investigative procedures in assessing the biomedical, sociodemographic, behavioral, physical, and psychological factors which contribute to the health and disease of the general population, with a special focus on multimorbidity and complex genetics.^27,28^ Data and DNA obtained from a first follow-up visit after 5 years were also available for analysis.^27,29^ The Lifelines cohort study was shown to be representative for the general population living in the Northern part of the Netherlands.^30^ The study was performed in compliance with the Declaration of Helsinki and approved by the medical ethical committee of the University Medical Center Groningen.

### Definition of thrombocytopenia, thrombocytosis, and other blood count abnormalities

Total and differential blood cell parameters were measured on a XE2100-system (Sysmex, Japan). As platelet counts decline and the prevalence of CH increases over aging, especially in individuals ≥60 years,^3,6,9,31^ we selected individuals with thrombocytopenia and thrombocytosis ≥60 years to study CH. Thrombocytopenia was defined as a platelet count <150 × 10^9^/L, and thrombocytosis by a platelet count >400 × 10^9^/L. We selected matched controls for each cohort by matching 1:1 for age and sex. All available biobanked peripheral blood samples from participants with thrombocytosis (n = 178), thrombocytopenia (n = 631), and their matched controls were obtained for targeted error-corrected next-generation sequencing (NGS). A concurrent cytopenia was defined as the presence of anemia (female, hemoglobin [Hb] <12.0 g/dL; male, Hb <13.0 g/dL)^7^ or neutropenia (neutrophil count <1.8 × 10^9^/L, local standard). A concurrent cytosis was defined as the presence of erythrocytosis (female, Hb >16.5 g/dL or hematocrit [Hct] ≥48%; male Hb >18.5 g/dL or Hct ≥52%)^16^ or leukocytosis (white blood cell [WBC] >10.0 × 10^9^/L, local standard).

### Targeted error-corrected NGS

A targeted error-corrected NGS panel covering 27 genes was used to study CH (Suppl. Table S1), as described earlier.^32^ Single-molecule molecular inversion probes were used to call somatic variants with a variant allele frequency (VAF) of at least 1% and with at least 10 consensus reads. All variants were inspected and manually curated to exclude recurrent artifacts and polymorphisms. The mean number of aligned consensus reads was 8470, with a coverage >500× for 97.6% of all targeted regions in the thrombocytopenia cohort (cases and controls) (Suppl. Figure S1). The mean number of aligned consensus reads was 8571 for the thrombocytosis cohort, with a coverage >500× for 97.8% of all targeted regions (Suppl. Figure S2).

### Statistical analysis

All statistical analyses were performed with R version 4.0.2. Two-group parametric data were statistically compared using the Student’s *t* test and nonparametric data using the Mann-Whitney *U* test. Categorical data were presented as absolute numbers and percentages. Differences between categorical groups were statistically tested using Fisher’s exact test. The Jonckheere-Terpstra test was used for assessing the increase in CH prevalence over age groups. Spearman’s rank correlation coefficient was used to assess correlation between the VAF and platelet counts (Suppl. methods).

The incidence of hematological malignancies was retrieved by linkage with the nationwide Netherlands Cancer Registry (NCR), which is maintained and hosted by the Netherlands Comprehensive Cancer Organization (IKNL). The NCR receives notifications of all newly diagnosed malignancies in the Netherlands since 1989 with nationwide coverage of at least 95%. All registered cancers are confirmed by histology and/or cytology. Participants were linked using pseudonyms based on the first 8 characters of their last name, date of birth, sex, and postal code at time of diagnosis. We used the International Classification of Diseases for Oncology morphology codes 9590-9999 for hematological malignancies. Time to malignancy was calculated from inclusion in the Lifelines study until a registered diagnosis of hematological malignancy. Individuals with a recorded prevalent malignancy were excluded from the analysis, so we only used incident cases. Data on cancer incidence were censored to December 2019, resulting in a median follow-up period of 7.5 years. Cumulative incidence graphs for incident diagnosis of hematological malignancies were constructed using the Aalen-Johansen estimator, with death as a competing risk and *P* values reported from Gray’s test.

The Kaplan-Meier estimator was used to visualize overall survival (OS). Survival time was defined in months from inclusion in Lifelines to death from any cause (derived from the Nationwide Population Registries Network, last updated in June 2020) or censoring. Log-rank tests were used for univariable comparison of OS. Cox proportional hazard regression was used to calculate the hazard ratios (HRs) with 95% confidence interval (CI) for risk of death, corrected for age and sex. HRs, unless reported otherwise, are derived from multivariable cox regression analysis. All statistical tests were performed two-sided and a *P* value below 0.05 was considered significant.

## RESULTS

### The emergence of thrombocytopenia, but not thrombocytosis, with aging

**Figure 1.**
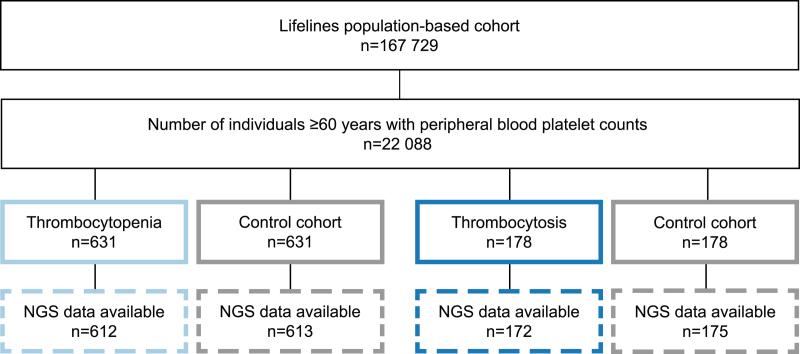
**Cohort overview and selection of cases for NGS.** Flowchart of the cohort selection process from the complete Lifelines cohort (n = 167,729). Thrombocytosis was defined as a peripheral blood platelet count >400 × 10^9^/L, and thrombocytopenia <150 × 10^9^/L. Controls were matched 1:1 for age and sex. NGS = next-generation sequencing.

We investigated all participants from the population-based Lifelines cohort ≥60 years with available peripheral platelet counts (n = 22,088). In total, 631 (2.9%) individuals had thrombocytopenia and 178 (0.8%) had thrombocytosis at study inclusion (Figure 1). In contrast to the prevalence of thrombocytopenia, which is known to increase with age in individuals ≥60 years,^9^ the prevalence of thrombocytosis remained stable upon aging (Suppl. Figure S3). Male predominance was observed in individuals with thrombocytopenia (78.6%) (Table 1; Suppl. Table S4), while a female predominance was observed in cases with thrombocytosis (75.3%) (Table 2; Suppl. Table S5), consistent with previously reported sex differences in platelet counts.^9^

**Table 1:** Characteristics and Peripheral Blood Counts of Community-dwelling Individuals ≥60 y With and Without Thrombocytopenia and 1:1 Matched Controls

	Thrombocytopenia N = 631	Absence of Thrombocytopenia N = 21,457	1:1 Matched Controls N = 631	N
Age (y)	67.0 [60.0; 88.0]	65.0 [60.0; 93.0]	67.0 [60.0; 88.0]	22,088
Male sex	496 (78.6%)	9597 (44.7%)	496 (78.6%)	22,088
Platelet count (10^9^/L)	131 (19.3)	243 (54.3)	230 (51.1)	22,088
Thrombocytopenia at follow-up	217 (34.4%)	178 (0.8%)	<10 (<1.6%)	16,555
Severe thrombocytopenia^a^	36 (5.7%)	0 (0.0%)	0 (0.0%)	22,088
WBC count (10^9^/L)	5.67 (6.01)	5.93 (1.88)	6.17 (1.85)	22,087
Neutrophil count (10^9^/L)	2.83 (0.93)	3.16 (1.10)	3.36 (1.12)	21,707
Hemoglobin concentration (g/dL)	14.8 (1.25)	14.2 (1.15)	14.6 (1.26)	22,087
Concurrent cytopenia^b^	97 (15.4%)	1870 (8.7%)	56 (8.9%)	21,707
Concurrent cytosis^c^	13 (2.1%)	464 (2.2%)	16 (2.5%)	21,707

Data were presented as mean (SD), median [min; max] for age and number (%) for categorical variables. N is the total number of evaluable individuals ≥60 y.

a Severe thrombocytopenia was defined as platelet count <100 × 10^9^/L.

b A concurrent cytopenia was defined as the presence of anemia (female, Hb <12.0 g/dL; male, Hb <13.0 g/dL) or neutropenia (neutrophil count <1.8 × 10^9^/L).

c A concurrent cytosis was defined as the presence of erythrocytosis (female, Hb >16.5 g/dL or Hct ≥48%; male Hb >18.5 g/dL or Hct ≥52%) or leukocytosis (WBC >10.0 × 10^9^/L).

Hb = hemoglobin; Hct = hematocrit; SD = standard deviation; WBC = white blood cell.

**Table 2:** Characteristics and Peripheral Blood Counts of Community-dwelling Individuals ≥60 y With and Without Thrombocytosis and 1:1 Matched Controls

	Thrombocytosis N = 178	Absence of Thrombocytosis N = 21,910	1:1 Matched Controls N = 178	N
Age (y)	65.0 [60.0; 87.0]	65.0 [60.0; 93.0]	65.0 [60.0; 87.0]	22,088
Male sex	44 (24.7%)	10049 (45.9%)	44 (24.7%)	22,088
Platelet count (10^9^/L)	475 (121.9)	238 (51.7)	248 (55.9)	22,088
Thrombocytosis at follow-up	71 (39.9%)	217 (1.0%)	<10 (<5.6%)	16,555
Severe thrombocytosis^a^	59 (33.1%)	0 (0.0%)	0 (0.0%)	22,088
White blood cell count (10^9^/L)	7.75 (2.08)	5.91 (2.11)	5.96 (1.61)	22,087
Neutrophil count (10^9^/L)	4.42 (1.63)	3.14 (1.09)	3.11 (1.13)	21,707
Hemoglobin concentration (g/dL)	13.4 (1.31)	14.2 (1.15)	14.0 (1.14)	22,087
Concurrent cytopenia^b^	27 (15.2%)	1920 (8.8%)	21 (11.8%)	21,707
Concurrent cytosis^c^	28 (15.7%)	445 (2.0%)	<10 (<5.6%)	21,707

Data were presented as mean (SD), median [min; max] for age and number (%) for categorical variables. N is the total number of evaluable individuals ≥60 y.

a Severe thrombocytosis was defined as platelet count >450 × 10^9^/L.

b A concurrent cytopenia was defined as the presence of anemia (female, Hb <12.0 g/dL; male, Hb <13.0 g/dL) or neutropenia (neutrophil count <1.8 × 10^9^/L).

c A concurrent cytosis was defined as the presence of erythrocytosis (female, Hb >16.5 g/dL or Hct ≥48%; male Hb >18.5 g/dL or Hct ≥52%) or leukocytosis (WBC >10.0 × 10^9^/L).

Hb = hemoglobin; Hct = hematocrit; SD = standard deviation; WBC = white blood cell.

### Thrombocytosis, but not thrombocytopenia, is associated with a higher prevalence of CH

**Figure 2.**
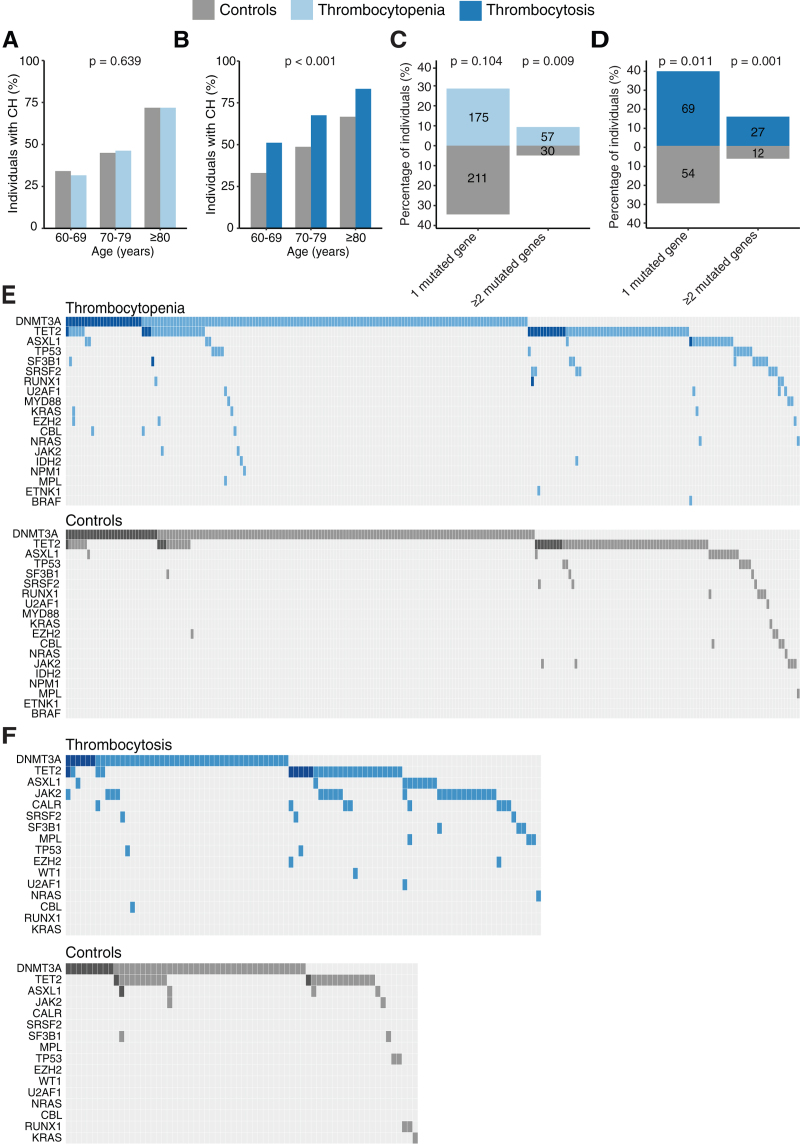
**Mutational patterns for individuals with thrombocytopenia/thrombocytosis and 1:1 matched controls.** (A) Prevalence of CH per age group for all individuals with thrombocytopenia (n = 612) and 1:1 matched controls (n = 613). The prevalence of CH significantly increased over age for both thrombocytopenia cases (*P* < 0.001) and their matched controls (*P* < 0.001). (B) Prevalence of CH per age group for all individuals with thrombocytosis (n = 172) and 1:1 matched controls (n = 175). The prevalence of CH significantly increased over age for both thrombocytosis cases (*P* = 0.030) and their matched controls (*P* = 0.031). (C) Proportion of individuals with 1 mutated gene and at least 2 mutated genes for individuals with thrombocytopenia and their 1:1 matched controls. (D) Proportion of individuals with 1 mutated gene and at least 2 mutated genes for individuals with thrombocytosis and their 1:1 matched controls. (E) The mutational landscape for individuals with thrombocytopenia (blue) and 1:1 matched controls (gray). (F) The mutational landscape for individuals with thrombocytosis (blue) and 1:1 matched controls (gray). Darker shades in panel (E) and (F) represent individuals with ≥2 mutations in the respective gene. CH = clonal hematopoiesis.

We performed sensitive targeted error-corrected sequencing for 27 hematological driver genes, to detect CH in individuals ≥60 years with thrombocytopenia or thrombocytosis and 1:1 matched controls (Suppl. Table S6, S7). With increasing age, as expected, the prevalence of CH increased in individuals with thrombocytopenia (*P* < 0.001; Figure 2A) and thrombocytosis (*P* = 0.030; Figure 2B), as well as in the controls. The prevalence of CH in cases with thrombocytopenia was comparable with their matched controls (37.9% vs 39.3%; *P* = 0.639), even when the definition of thrombocytopenia was lowered from <150 to <100 × 10^9^/L (Suppl. Figure S4). In contrast, an increased prevalence of CH, across all age groups, was observed in individuals with thrombocytosis (55.8% vs 37.7%; *P* < 0.001). If the definition of thrombocytosis was increased from >400 to >450 × 10^9^/L an even higher number of individuals with CH was observed compared with their matched controls (71.4% vs 32.1%; *P* < 0.001; Suppl. Figure S4). The proportion of cases with multiple mutated genes was increased in individuals with thrombocytopenia (*P* = 0.009) and thrombocytosis (*P* = 0.001), as compared with their matched controls (Figure 2C,D).

Overall, 354 somatic mutations in 19 different genes were identified in 612 individuals with thrombocytopenia and 141 somatic mutations in 14 different genes were identified in 172 individuals with thrombocytosis (Figure 2E,F; Suppl. Tables S2, S3). Mutations in genes associated with MPNs (JAK2, CALR, and MPL) were identified in 32 of 172 (18.6%) individuals with thrombocytosis versus 2 of 175 (1.1%) in their matched controls. The mutational landscapes of cases with thrombocytopenia or thrombocytosis and their matched controls also illustrate that, although the landscapes look comparable, the individuals with abnormal platelet counts have more comutations (Figure 2E,F; Suppl. Figure S5,S6). In addition, the cases with thrombocytopenia and thrombocytosis carry more different combinations of gene mutations compared with to their matched controls (Suppl. Figure S7).

### Specific mutational patterns are associated with platelet count abnormalities

**Figure 3.**
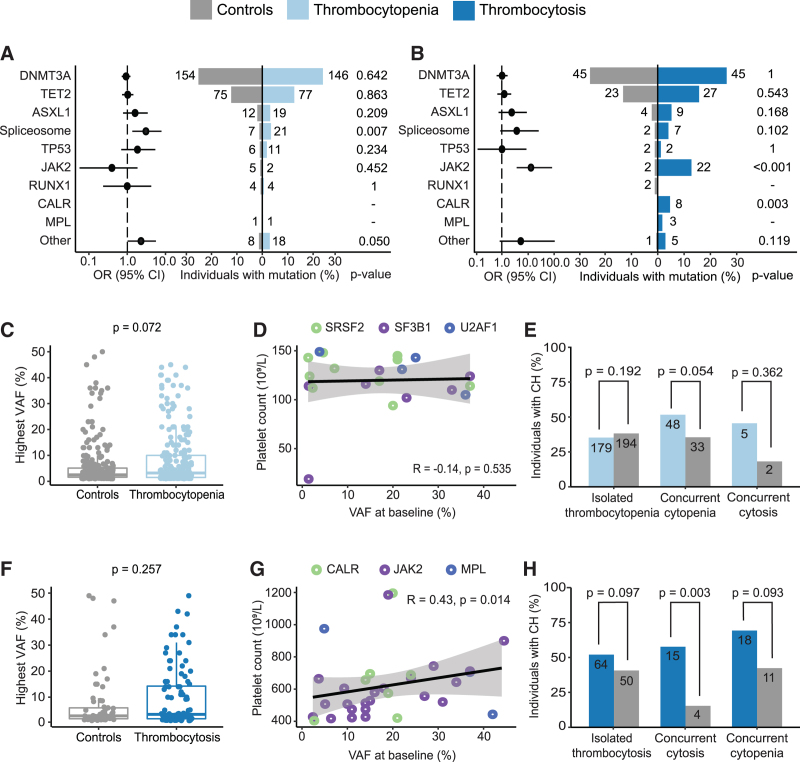
**Clonal hematopoiesis-associated mutations in cases with thrombocytopenia/thrombocytosis and 1:1 matched controls.** (A) Pyramid plot indicating the proportion of individuals with mutations in the most commonly mutated genes for individuals with thrombocytopenia (light blue) and 1:1 matched controls (gray). (B) Pyramid plot indicating the proportion of individuals with mutations in the most commonly mutated genes for individuals with thrombocytosis (dark blue) and 1:1 matched controls (gray). (C) Distribution for the maximum VAF detected in all individuals with thrombocytopenia and 1:1 matched controls. (D) Relation between VAF (%) of spliceosome mutations and the absolute platelet count (10^9^/L) in individuals with thrombocytopenia. (E) Prevalence of CH for individuals with isolated thrombocytopenia vs thrombocytopenia with concurrent cytopenia or cytosis, compared with their respective matched controls. (F) Distribution for the maximum VAF detected in all individuals with thrombocytosis and 1:1 matched controls. (G) Relation between the VAF (%) of *JAK2*/*CALR/MPL* mutations and the absolute platelet count (10^9^/L) in individuals with thrombocytosis. (H) Prevalence of CH for isolated thrombocytosis vs thrombocytosis with a concurrent cytopenia or cytosis compared with respective matched controls. Spliceosome mutations include *SF3B1, SRSF2* and *U2AF1.* CH = clonal hematopoiesis; OR = odds ratio; VAF = variant allele frequency.

To identify specific mutational patterns associated with thrombocytopenia or thrombocytosis, we compared the prevalence of the mutated genes (Figure 3A,B; Suppl. Figure S8). Spliceosome mutations (*SF3B1*, *SRSF2,* and *U2AF1*) were enriched in individuals with thrombocytopenia compared with their matched controls (3.4% vs 1.1%; *P* = 0.007; Figure 3A). Individuals with thrombocytosis, compared with their matched controls, had a higher prevalence of *JAK2* (12.8% vs 1.1%; *P* < 0.001) (all V617F mutations) and *CALR* mutations (4.7% vs 0.0%; *P* = 0.003; Figure 3B). Clone sizes were not significantly different for cases with thrombocytopenia compared with their matched controls (median VAF 3.3% vs 2.6%; *P* = 0.072) or thrombocytosis compared with their matched controls (median VAF 3.6% vs 2.9%; *P* = 0.257) (Figure 3C,F). No correlation between the VAF of spliceosome-associated genes and platelet counts was observed (Figure 3D; Suppl. Figure S10). The clone sizes of MPN-associated mutations (*JAK2, CALR*, or *MPL*) were positively associated with the absolute number of peripheral blood platelet counts (Spearman rank correlation coefficient 0.43; *P* = 0.014; Figure 3G; Suppl. Figure S11). The VAF of *DNMT3A* mutations was significantly lower in thrombocytosis cases compared with their matched controls (*P* = 0.018; Suppl. Figure S9B), but no significant correlation between the VAF of *DNMT3A* mutations and platelet counts was observed (Suppl. Figure S11B). An increased proportion of MPN-associated driver mutations (*JAK2*, *CALR*, *MPL*) was observed in individuals with more severe thrombocytosis (ie, platelet count >450 × 10^9^/L) (44.6% vs 0.0%; *P* < 0.001), but not for those with mild thrombocytosis (ie, platelet count 400–450 × 10^9^/L; 6.0% vs 1.7%; *P* = 0.171; Suppl. Figure S6) compared with their matched controls. A recent study in ET patients has shown that *JAK2-V617F* mutations cause increased systemic inflammatory levels,^33^ but high-sensitivity C-reactive protein (hsCRP) levels were not significantly increased in thrombocytosis cases carrying a *JAK2-V617F* mutation compared with those without a *JAK2-V617F* mutation in our population-based cohort (*P* = 0.083).

### Prevalence of CH in thrombocytopenia and thrombocytosis cases with an additional cytopenia or cytosis

As individuals with abnormal platelet counts in combination with abnormal blood cell counts in another lineage may represent a specific subgroup, we studied the mutational spectrum in these cases. Increased mortality risks were already reported for thrombocytopenia cases in combination with anemia or neutropenia, without stratification for CH.^9^ In our cohort, 97 individuals (15.4%) with thrombocytopenia had a concurrent cytopenia, and 13 (2.1%) a concurrent cytosis (Table 1; Suppl. Table S4; Suppl. Figure S13A,B). The prevalence of CH in cases with isolated thrombocytopenia or with an additional cytopenia/cytosis was not significantly different compared with their matched controls (Figure 3E). Significantly larger clones were observed in individuals with thrombocytopenia and a concurrent cytopenia compared with their matched controls (*P* = 0.019; Suppl. Figure S12C), whereas the average clone size was comparable between cases with isolated thrombocytopenia compared with their matched controls. Due to low numbers, clone size in individuals with thrombocytopenia and another cytosis was not evaluated. After correction for age and sex, the presence of CH did not affect survival in individuals with thrombocytopenia and an additional cytopenia (*P* = 0.430; Suppl. Table S12A).

In cases with thrombocytosis, 28 individuals (15.7%) had a concurrent cytosis, whereas 27 cases (15.2%) carried a concurrent cytopenia (Table 2; Suppl. Table S5; Suppl. Figure S13C). The prevalence of CH was not significantly increased in isolated thrombocytosis cases compared with their matched controls (52.0% vs 40.7%; *P* = 0.097), but was significantly increased in individuals with thrombocytosis and a concurrent cytosis compared with their matched controls (57.7% vs 15.4%; *P* = 0.003; Figure 3H). *JAK2* mutations were enriched in both cases with isolated thrombocytosis (10.3% vs 1.4%; *P* = 0.002) as well as in cases carrying an additional cytosis (26.9% vs 0.0%; *P* = 0.010) compared with their respective matched controls (Suppl. Figure S12F). The OS of individuals with thrombocytosis in combination with an additional cytosis was not affected after a median follow-up time of 94 months (*P* = 0.554; Suppl. Table S12B).

### Persistence of platelet count abnormalities over time and its relation with CH

**Figure 4.**
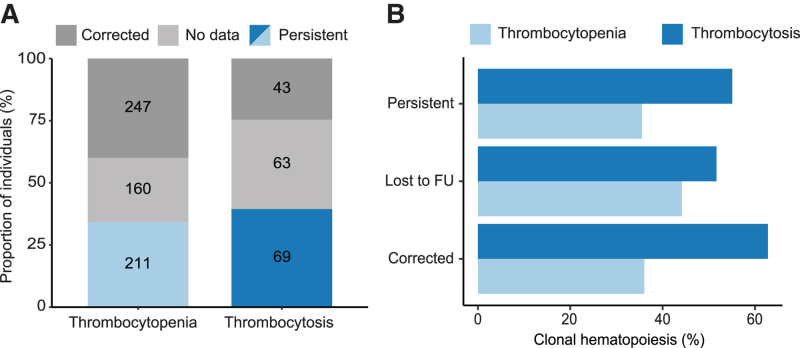
**Follow-up for individuals with platelet count abnormalities.** (A) Proportion of individuals with persistent thrombocytosis (dark blue) and thrombocytopenia (light blue), transient thrombocytosis and thrombocytopenia (dark gray) and the proportion of individuals without data at follow-up (light gray). The follow-up time was approximately 5 y. (B) Proportion of individuals carrying clonal hematopoiesis in persistent cases, corrected cases, and the individuals lost at follow-up. FU = follow-up.

Data from follow-up visit were available for 458 individuals with thrombocytopenia and 112 with thrombocytosis after a median follow-up time of 94 months (range 5–136 months). Persistent thrombocytopenia at follow-up (ie, persistent platelet count <150 × 10^9^/L) was observed in 211 of 458 (46.1%) cases (Figure 4A; Suppl. Figure S14A). Persistent thrombocytosis (platelet count >400 × 10^9^/L) was observed for 69 of 112 (61.6%) individuals at follow-up (Figure 4A; Suppl. Figure S14B). Among all controls, 12 individuals developed thrombocytopenia at follow-up and 15 developed thrombocytosis.

The prevalence of CH in cases with persistent thrombocytopenia was comparable (35.5%) to the prevalence of CH in individuals with transient thrombocytopenia (36.0%; *P* = 0.922; Figure 4B). The CH prevalence did also not significantly differ between individuals with persistent thrombocytosis (55.1%) compared with cases with transient thrombocytosis (62.8%; *P* = 0.439; Figure 4B). *JAK2* mutations were observed in a large proportion of cases with persistent thrombocytosis (11/38, 28.9%) (Suppl. Figure S15C, D), while mutations in *JAK2* were only observed in 3 of 27 (11.1%) individuals with transient thrombocytosis. In addition, we found significantly higher hsCRP levels in transient thrombocytosis cases compared with persistent thrombocytosis cases (median 6.0 vs 2.2; *P* = 0.006). In total, 11 of 14 individuals with thrombocytosis that carried a *JAK2* mutation at baseline with available follow-up data had persistent thrombocytosis (78.6%). The prevalence of CH was not increased in individuals that were lost at follow-up for any reason (Figure 4B).

### Inferior survival for cases with thrombocytopenia in combination with multiple mutated genes or mutations in *TP53* or spliceosome genes

**Figure 5.**
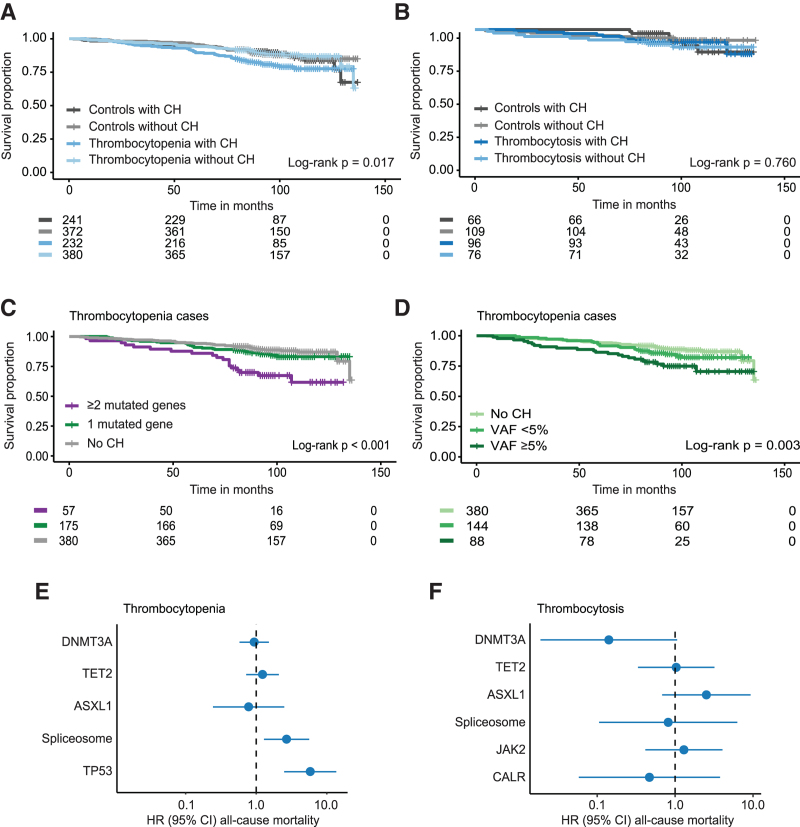
**Prediction of all-cause mortality by CH and mutational patterns for individuals with platelet count abnormalities.** (A) Kaplan-Meier curve for OS of individuals with thrombocytopenia with or without CH and 1:1 matched controls with or without CH. The displayed *P* value was derived from a log-rank test, which is not corrected for age and sex. (B) Kaplan-Meier curve for OS of individuals with thrombocytosis with or without CH and 1:1 matched controls with or without CH. (C) Kaplan-Meier curve representing OS of thrombocytopenia cases stratified by the number of mutated genes. (D) Kaplan-Meier curve representing OS of thrombocytopenia cases stratified by the maximum detected clone size. (E) Forest plot to show the risk of all-cause mortality associated with mutations in specific genes in individuals with thrombocytopenia. Cox proportional hazard regression included age and sex as covariates. Absence of mutations in the respective genes was used as a reference. We restricted to genes with ≥5 mutations. (F) Forest plot to show the risk of all-cause mortality associated with mutations in specific genes in individuals with thrombocytosis. Cox proportional hazard regression included age and sex as covariates. Absence of mutations in the respective genes was used as a reference. Analyses were restricted to genes with ≥5 detected mutations. CI = confidence interval; CH = clonal hematopoiesis; HR = hazard ratio; OS = overall survival; VAF = variant allele frequency.

As CH is associated with an increased all-cause mortality, we investigated the survival of individuals with thrombocytopenia or thrombocytosis and CH. Individuals with thrombocytopenia and CH did not have a significantly worse OS compared with cases with thrombocytopenia without CH after correction for age and sex (Figure 5A; Suppl. Table S8A). Thrombocytopenia cases that carried multiple mutated genes showed inferior OS (HR = 2.08, 95% CI, 1.24-3.50; *P* = 0.006), compared with those without CH or with CH and a single-mutated gene (Figure 5C; Suppl. Table S9A). The effect of multiple mutated genes on survival was not present in the matched controls (Suppl. Figure S16). In addition, individuals with thrombocytopenia and CH with larger clones (VAF ≥5%) had a poor survival compared with those without CH or with smaller clone sizes, but this effect was lost after correction for age and sex (HR = 1.56, 95% CI, 0.95-2.55; *P* = 0.079; Figure 5D; Suppl. Table S10A). Subsequently, we investigated the impact of the most frequently mutated genes in individuals with thrombocytopenia on survival. This revealed that the presence of a mutation in *TP53* (HR = 5.83, 95% CI, 2.49-13.64; *P* < 0.001) or in one of the spliceosome-associated genes (HR = 2.69, 95% CI, 1.29-5.63; *P* = 0.009) significantly increased the risk of death for individuals with thrombocytopenia (Figure 5E; Suppl. Table S11A).

The presence of thrombocytosis did not impact OS, neither with nor without CH after correction for age and sex (Figure 5B; Suppl. Figure S16). In addition, specific gene mutations, including those in MPN driver genes, did not impact OS in individuals with thrombocytosis (Figure 5F; Suppl. Table S11B). However, a near-significant protective effect of *DNMT3A* mutations on the survival of thrombocytosis cases was found (*P* = 0.057; Figure 5F).

### A higher cumulative incidence of hematological malignancies in cases with thrombocytosis and CH, but not for cases with thrombocytopenia

**Figure 6.**
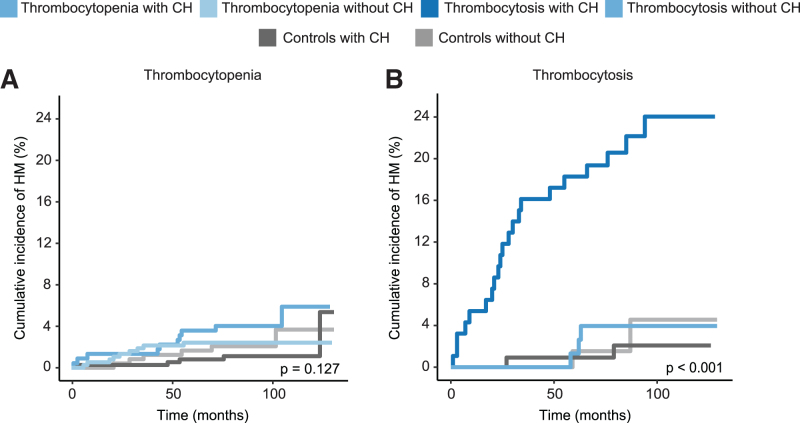
**Incident diagnosis of hematological malignancies.** (A) Cumulative incidence for diagnosis of hematological malignancies in individuals with thrombocytopenia and controls, stratified by the presence of CH. (B) Cumulative incidence for diagnosis of hematological malignancies in individuals with thrombocytosis and controls, stratified for the presence of CH. CH = clonal hematopoiesis; HM = hematological malignancies.

As CH is associated with an increased risk to develop hematological malignancies, we investigated incident diagnoses of hematological malignancies by establishing linkage of the Lifelines cohort to the nationwide population-based NCR. Although the numbers were low, 10 individuals with thrombocytopenia and CH were diagnosed with hematological malignancies during follow-up (4.5% of the cases). The incidence of newly diagnosed hematological malignancies was comparable among cases with thrombocytopenia with and without CH and their matched controls with and without CH (*P* = 0.127; Figure 6A; Suppl. Table 13A).

In contrast, individuals with thrombocytosis and CH had a high cumulative incidence of hematological malignancies (21 of 93, estimated incidence 18.3% after 5 years, 95% CI, 10.4%-26.2%; Figure 6B; Suppl. Table 13B). From these, 17 cases were diagnosed with MPN. MPN-associated mutations at Lifelines inclusion were detected for 16 of these cases: 11 individuals carrying a mutation in *JAK2*, 5 carrying *CALR* mutations, and 1 carrying a *MPL* mutation.

## DISCUSSION

We used a case–control study design in a large community-dwelling cohort to gain insight into the association between CH and platelet count abnormalities. Compared with matched controls, a higher frequency of CH was found for individuals with thrombocytosis, but not for individuals with thrombocytopenia. Mutations in spliceosome-associated genes were enriched in cases with thrombocytopenia compared with matched controls, whereas MPN-associated mutations (*JAK2*, *CALR*, *MPL*) were enriched in cases with thrombocytosis. Our data indicate that, in contrast to genes that are frequently mutated with aging (*DNMT3A* and *TET2*), specific gene mutations have clinical relevance in the context of these platelet count abnormalities.

Clonal gene mutations in the presence of disturbed platelet counts may suggest a premalignant stage of myeloid disorders including MDS and MPN. Earlier published studies showed an association between CH and an increased risk of hematologic cancer in population-based individuals.^3,4^ Overall, we did not observe a higher incidence of hematological malignancies in cases with thrombocytopenia and CH, compared with age- and sex-matched controls with CH. This suggests that overall, the combination of CH and thrombocytopenia in community-dwelling individuals is not indicative of early myeloid dysplasia.

Although the combination of thrombocytopenia and general CH is not indicative of early myeloid dysplasia, we found specific mutational patterns that might be of clinical interest. Mutations involved in the spliceosome complex (*SF3B1*, *SRSF2*, *U2AF1*), which are commonly mutated in MDS patients, were significantly enriched in population-based individuals with thrombocytopenia.^34,35^ Similarly, spliceosome mutations were enriched in older individuals with anemia compared with matched controls.^7^ However, the enrichment of spliceosome mutations in thrombocytopenia cases could not be explained by the presence of additional cytopenias, for instance anemia. Spliceosome defects are strongly associated with the development of myeloid malignancies, suggesting that these individuals are at risk of (early) clonal disturbances that might lead to oncogenic transformation.^36,37^ This is in line with an increased risk of progression to myeloid disease observed for patients presenting with CCUS in clinical practice.^38^ In our cohort, we did not observe a significant enrichment of hematological malignancies in individuals with thrombocytopenia that carry spliceosome mutations, but this is presumably explained by the small number of individuals (n = 21). In addition, most published data on CCUS are based on individuals who clinically present with hematological problems, which is different from individuals who are identified in a nonclinical population-based cohort like ours.^38,39^

Cases with thrombocytopenia carried more frequently multiple mutated genes that associated with an increased risk of death. A higher number of mutated genes is also associated with a worse prognosis in MDS patients.^7,34,35^ These mutational patterns deviating from age-related CH might represent (premalignant) clonally disturbed hematopoiesis and these cases are at risk for the presence or development of myeloid malignancies. Selective pressures in the bone marrow niche of cases with thrombocytopenia may increase the chance of acquiring secondary mutations.^40^ However, mechanistic studies are needed to investigate the pathophysiologic relevance of combinations of clonal gene mutations on malignant transformation.

Furthermore, mutations in *TP53* increased the risk of death in individuals with thrombocytopenia. This effect was not observed in the oldest old (80+) Lifelines participants or in cases with anemia, monocytosis, or erythrocytosis that carried *TP53* mutations from the Lifelines population-based cohort, and seems therefore specific for individuals with thrombocytopenia.^7,16,23,32^ We identified 10 of 11 *TP53* mutations in cases with isolated thrombocytopenia. A recent study showed that monoallelic *TP53* mutations in patients with MDS are not independently predictive of inferior survival.^41^ Although we cannot exclude loss of heterozygosity in the other allele, we did not detect multiple mutations in *TP53* in these individuals. The mutational spectrum of *TP53* cases included in our study is less complex than in cases with MDS, which may explain differences in the pathophysiology of a *TP53* mutation in thrombocytopenia cases compared with MDS cases. Studies in mice and human cells showed that *TP53* knockout or *TP53* knockdown cause functional platelet defects. In a large cohort of multiple myeloma cases, homozygous deletion of *TP53* was associated with decreased platelet counts.^42,43^ Although further research is required, these data suggest that *TP53* mutations directly affect platelet development.

Mutations in MPN-associated genes *JAK2*, *CALR* and *MPL* were observed at considerably high frequency in individuals with thrombocytosis. Indeed, 12.8% of the cases with thrombocytosis carried a *JAK2* mutation. This percentage increased to 26.9% of the individuals when the thrombocytosis was accompanied by an additional cytosis. The low frequency of *JAK2* clones among matched controls without blood count abnormalities was in agreement with three Danish population-based studies.^24,44,45^ The severity of thrombocytosis correlated with the clone size of MPN-associated genes in our cohort and *JAK2* mutant cases were likely to have persistent thrombocytosis (78.6%). Recent studies showed that ET patients with a low *JAK2* mutational burden have enrichment of the clone within the megakaryocyte compartment.^46^ As hsCRP levels were significantly higher in transient thrombocytosis cases, it is very likely that a proportion of these cases can be explained by systemic inflammation. Studies in ET patients have shown that *JAK2-V617F* mutations cause increased systemic inflammatory levels, potentially directly enhancing the clonal expansion. We did not observe a significant increase in hsCRP levels in thrombocytosis cases with *JAK2-V617F* mutations compared with cases without mutant *JAK2*, but this may likely be explained by the smaller clone sizes in our cohort compared with MPN cases.^33^

The presence of CH in cases with thrombocytosis did not impact OS, illustrating the favorable prognosis for patients diagnosed with ET.^47,48^ However, we found a near-significant protective effect of *DNMT3A* mutations on OS in thrombocytosis cases. A similar effect was previously observed in the context of stem cell transplantations, as patients that received *DNMT3A* mutated stem cells showed increased survival and better engraftment of their stem cells.^49^ It is unclear how *DNMT3A* mutations positively affect the survival of thrombocytosis cases, but this seems at the root of an interesting phenomenon that has been observed more frequently in the context of deregulated hematopoiesis.

In our cohort, with a robust linkage with the Dutch cancer registry, we found a significantly higher incidence of hematological malignancies in cases with thrombocytosis and CH. A total of 48.5% of *JAK2*/*MPL*/*CALR* mutant cases with thrombocytosis were diagnosed with a hematological malignancy at follow-up. We may conclude that the large proportion of community-based individuals with thrombocytosis in combination with *JAK2*/*MPL*/*CALR* mutations represented premalignant or underdiagnosed cases of MPN.

In conclusion, we identified mutational patterns of clinical relevance when detected in community-dwelling cases with thrombocytopenia and thrombocytosis. In combination with multiple mutated genes or *TP53*/spliceosome mutations, individuals with thrombocytopenia have an increased risk of death. The combination of thrombocytosis and *JAK2*/*MPL*/*CALR* mutations is highly suggestive for undiagnosed MPN or a premalignant condition, while other gene mutations seem to have no relevance in these cases.

## ACKNOWLEDGMENTS

Our gratitude goes out to all the participants of the Lifelines cohort study. The authors thank the registration team of the Netherlands Comprehensive Cancer Organisation (NCR) for the collection of data for the Netherlands Cancer Registry as well as IKNL staff for scientific advice.

## AUTHOR CONTRIBUTIONS

PK, MGJMvB, IAvZ, AOdG, and JBS contributed to study design, data collection, analysis and interpretation of the data; JJS, AGD, and BAvdR were involved in the interpretation of the data; GH and JHJ were principal investigators and involved in the study design, data collection, and interpretation of the results; PK and MGJMvB wrote the manuscript that was critically revised by all coauthors.

## DISCLOSURES

The authors have no conflicts of interest to disclose.

## SOURCES OF FUNDING

This work was supported by the MDS-RIGHT project, which has received funding from the European Union’s Horizon 2020 research and innovation program under grant agreement 634789. The funder of this study had no role in study design, collection, analysis, and interpretation of data, and writing or approval of the manuscript. The Lifelines Biobank initiative has been made possible by subsidy from the Dutch Ministry of Health, Welfare and Sport; the Dutch Ministry of Economic Affairs; the University Medical Center Groningen; University Groningen; and the Northern Provinces of The Netherlands. AOdG and JHJ were supported by a grant from the Dutch Cancer Society (grant number 10813).

## Supplementary Material
