# Supplementary material for: Abnormal Platelet Counts and Clonal Hematopoiesis in the General Population
Source: Hemasphere. 2023 Jan 5;7(1):e821. doi: 10.1097/HS9.0000000000000821 (PMC9829283; doi:10.1097/HS9.0000000000000821)

Supplemental Digital Content (SDC)

Supplement to: Abnormal platelet counts and clonal hematopoiesis in the general population. Priscilla Kamphuis, Maaike G.J.M. van Bergen, Isabelle A. van Zeventer, Aniek O. de Graaf, Avinash G. Dinmohamed, Jonas B. Salzbrunn, Jan Jacob Schuringa, Bert A. van der Reijden, Gerwin Huls, Joop H. Jansen.

Table of contents

Supplemental Materials and Methods 3
Supplemental Tables 4
Supplemental Table 1 – Overview of sequenced genes and regions 4
Supplemental Table 2 – Frequencies of detected mutations in thrombocytopenia and control cohort 5
Supplemental Table 3 – Frequencies of detected mutations in thrombocytosis and control cohort 6
Supplemental Table 4 – Peripheral blood counts and baseline characteristics of the thrombocytopenia cohort 7
Supplemental Table 5 – Peripheral blood counts and baseline characteristics of the thrombocytosis cohort 8
Supplemental Table 6 – Baseline characteristics for thrombocytopenia and control cohort according to the presence of CH 9
Supplemental Table 7 – Baseline characteristics for thrombocytosis and control cohort according to the presence of CH 10
Supplemental Table 8 – Univariable and multivariable associations of clonal hematopoiesis with overall survival in individuals with platelet count abnormalities 11
Supplemental Table 9 – Univariable and multivariable associations for the number of mutated genes with overall survival in individuals with platelet count abnormalities 12
Supplemental Table 10 – Univariable and multivariable associations of clone size with overall survival for individuals with platelet count abnormalities 13
Supplemental Table 11 – Univariable and multivariable associations of specific gene mutations with overall survival for individuals with platelet count abnormalities 14
Supplemental Table 12 – Univariable and multivariable associations of clonal hematopoiesis with overall survival in isolated thrombocytopenia/thrombocytosis or with a concurrent cytopenia/cytosis 15
Supplemental Table 13 – Incident diagnoses of hematological malignancies 16
Supplemental Figures 17
Supplemental Figure 1 – Coverage in thrombocytopenia cohort 17
Supplemental Figure 2 – Coverage in thrombocytosis cohort 18
Supplemental Figure 3 – Prevalence of thrombocytosis in the population-based Lifelines cohort ≥60 years 19
Supplemental Figure 4 – Mutational spectrum for mild and severe thrombocytopenia or thrombocytosis 20
Supplemental Figure 5 – Mutational landscapes for mild and severe thrombocytopenia 21
Supplemental Figure 6 – Mutational landscapes for mild and severe thrombocytosis 22
Supplemental Figure 7 – Co-mutational patterns in individuals with platelet count abnormalities and their matched controls 23
Supplemental Figure 8 – Pyramid plots of all detected gene mutations 24
Supplemental Figure 9 – Variant allele frequency distribution per gene 25
Supplemental Figure 10 – Relation between VAF of commonly mutated genes and absolute platelet count for individuals with thrombocytopenia 26
Supplemental Figure 11 – Relation between VAF of commonly mutated genes and absolute platelet count for individuals with thrombocytosis 27
Supplemental Figure 12 – Mutational spectrum for isolated thrombocytopenia/thrombocytosis or with concurrent cytopenia/cytosis 28
Supplemental Figure 13 – Mutational landscapes for thrombocytopenia with concurrent cytopenia and thrombocytosis with concurrent cytosis 29
Supplemental Figure 14 – Evolution of platelet counts over time 30
Supplemental Figure 15 – Mutational landscapes for persistent and corrected thrombocytopenia and thrombocytosis 31
Supplemental Figure 16 – Overall survival stratified to the number of mutated genes and variant allele frequency in individuals with platelet count abnormalities 32

Supplemental Materials and methods

Statistical analyses

All statistical analyses were performed with R version 4.0.2. For data reshaping and summarization, we used the R packages dplyr v1.0.7 (https://github.com/tidyverse/dplyr/), tidyr v1.1.4 (https://github.com/tidyverse/tidyr), stringr v1.4.0 (https://github.com/tidyverse/stringr) and data.table v1.14.2 (https://github.com/Rdatatable/data.table). Visualizations were made using ggplot2 v3.3.5 (https://github.com/tidyverse/ggplot2) and RColorBrewer v1.1-2 (https://github.com/cran/RColorBrewer). Chord diagrams to show co-mutational patterns were made using the R package circlize v0.4.13 (https://github.com/jokergoo/circlize). For making the table with baseline characteristics of both cohorts R package CompareGroups v4.5.1 (https://github.com/isubirana/compareGroups) was used. Continuous data are presented as mean (SD) for parametric variables of median (range) for non-parametric variables. Categorical variables are displayed as absolute numbers and percentages. The student's t-test and Mann-whitney U test were used for statistical comparison of continuous parametric and non-parametric data respectively. Differences between categorical groups were statistically tested using Fisher's exact test and for testing the increase in CH prevalence over age groups the Jonckheere-Terpstra trend test in R package DescTools v0.99.44 (https://github.com/cran/DescTools) was used. The Spearman's rank correlation coefficient was used to assess correlation between the VAF and platelet counts. Odds ratios and confidence intervals were derived from univariable logistic regression.

Overall survival (OS) of participants was obtained from the Municipal Persons Records Database, with last consultation at June 2020. Survival analyses and visualizations were performed with R packages survival v3.2-13 (https://github.com/therneau/survival) and survminer v0.4.9 (https://github.com/kassambara/survminer).

All statistical tests were performed two-sided and a p-value below 0.05 was considered significant.

Supplemental Table 1 – Overview of sequenced genes and regions

Gene	Reference transcript	ENSEMBL reference transcript	Exon	Targeted codons/region
**ASXL1**	NM_015338	ENST00000375687	13 (partially)	exon 13
**BRAF**	NM_004333.4	ENST00000288602	15 (partially)	codon 600
**CALR**	NM_004343	ENST00000316448	9	exon 9
**CBL**	NM_005188	ENST00000264033	8-9	exon 8 and 9
**CSF3R**	NM_156039	ENST00000373103	14, 17	codon 618, 615 and exon 17
**DNMT3A**	NM_175629	ENST00000264709	2-23 (all coding exons)	all coding exons
**ETNK1**	NM_018638	ENST00000266517	3 (partially)	codon 243-244
**EZH2**	NM_004456	ENST00000320356	2-20 (all coding exons)	all coding exons
**FLT3_835**	NM_004119	ENST00000241453	20 (partially)	codon 835-842
**IDH1**	NM_005896	ENST00000415913	4 (partially)	codon 132
**IDH2**	NM_002168	ENST00000330062	4 (partially)	codon 140, 172
**JAK2**	NM_004972	ENST00000381652	12, 14 (partially)	codon 617 and exon 12
**KIT**	NM_000222	ENST00000288135	8 (partially), 17 (partially)	codon 816, 419
**KRAS**	NM_004985	ENST00000256078	2-3 (partially)	a.o. codon 12, 13, 61
**MPL**	NM_005373	ENST00000372470	10 (partially)	codon 515, 505
**MYD88**	NM_002468.4	ENST00000417037	4-5 (partially)	codon 265 and 232
**NOTCH1**	NM_017617.4	ENST00000277541	34 (partially)	codon 2514
**NPM1**	NM_002520	ENST00000517671	11 (partially)	codon 288-290
**NRAS**	NM_002524	ENST00000369535	2-3 (partially)	a.o. codon 12, 13, 61
**RUNX1**	NM_001754	ENST00000437180	2-9 (all coding exons)	all coding exons
**SETBP1**	NM_015559	ENST00000282030	4 (partially)	codon 850-910
**SF3B1**	NM_012433	ENST00000335508	13-16	codon 575-790
**SRSF2**	NM_003016	ENST00000392485	1 (partially)	codon 95, 96
**TET2**	NM_001127208	ENST00000380013	3-11 (all coding exons)	all coding exons
**TP53**	NM_000546	ENST00000269305	2-11 (all coding exons)	all coding exons
**U2AF1**	NM_006758	ENST00000291552	2, 6 (partially)	codon 34, 157
**WT1**	NM_024426	ENST00000332351	7, 9	exon 7 and 9

Supplemental Table 2 – Frequencies of detected mutations in thrombocytopenia and control cohort

Frequencies for all detected somatic mutations for the thrombocytopenia cohort are shown in the table below. Some individuals carried multiple mutations within one gene, as indicated. Number of mutations and number of individuals with ≥2 mutations are given for the entire cohort including thrombocytopenia cases and their matched controls.

**Gene**	**Number of mutations**	**Number of individuals with ≥1 mutation**			**Number of individuals with ≥2 mutations**
		Entire cohort	Thrombocytopenia cohort (n=612)	Control cohort (n=613)	
DNMT3A	362	300	146	154	54
TET2	196	152	77	75	29
ASXL1	32	31	19	12	1
TP53	17	17	11	6	0
JAK2	7	7	2	5	0
SF3B1	14	13	10	3	1
SRSF2	10	10	7	3	0
CBL	6	6	3	3	0
BRAF	1	1	1	0	0
CALR	0	0	0	0	0
CSF3R	0	0	0	0	0
ETNK1	1	1	1	0	0
EZH2	6	6	3	3	0
FLT3	0	0	0	0	0
IDH1	0	0	0	0	0
IDH2	2	2	2	0	0
KIT	0	0	0	0	0
KRAS	4	4	3	1	0
MPL	2	2	1	1	0
MYD88	3	3	3	0	0
NOTCH1	0	0	0	0	0
NPM1	1	1	1	0	0
NRAS	3	3	2	1	0
RUNX1	9	8	4	4	1
SETBP1	0	0	0	0	0
U2AF1	5	5	4	1	0
WT1	0	0	0	0	0

Supplemental Table 3 – Frequencies of detected mutations in thrombocytosis and control cohort

Frequencies for all detected somatic mutations for the thrombocytosis cohort are shown in the table below. Some individuals carried multiple mutations within one gene, as indicated. Number of mutations and number of individuals with ≥2 mutations are given for the entire cohort including thrombocytosis cases and their matched controls.

Gene	Number of mutations	Number of individuals with ≥1 mutation			Number of individuals with ≥2 mutations
		Entire cohort	Thrombocytosis cohort (n=172)	Control cohort (n=175)	
DNMT3A	108	90	45	45	15
TET2	58	50	27	23	8
ASXL1	14	13	9	4	1
TP53	4	4	2	2	0
JAK2	24	24	22	2	0
SF3B1	5	5	3	2	0
SRSF2	3	3	3	0	0
CBL	1	1	1	0	0
BRAF	0	0	0	0	0
CALR	8	8	8	0	0
CSF3R	0	0	0	0	0
ETNK1	0	0	0	0	0
EZH2	2	2	2	0	0
FLT3	0	0	0	0	0
IDH1	0	0	0	0	0
IDH2	0	0	0	0	0
KIT	0	0	0	0	0
KRAS	1	1	0	1	0
MPL	3	3	3	0	0
MYD88	0	0	0	0	0
NOTCH1	0	0	0	0	0
NPM1	0	0	0	0	0
NRAS	1	1	1	0	0
RUNX1	2	2	0	2	0
SETBP1	0	0	0	0	0
U2AF1	1	1	1	0	0
WT1	1	1	1	0	0

Supplemental Table 4 – Peripheral blood counts and baseline characteristics of the thrombocytopenia cohort

A summary of baseline clinical characteristics and laboratory values for the thrombocytopenia cases, 1:1 matched control cohort and all other individuals ≥60 years with peripheral blood platelet counts that are not in the cohort. Data are presented as median [min;max] for continuous variables and absolute number (%) for categorical variables. Significance was tested for thrombocytopenia cases and their matched controls. A p-value below <0.05 is considered as significantly different. [1]A severe thrombocytopenia was defined as platelet count <100 x10^9/L. [2]A concurrent cytopenia was defined as the presence of anemia (female <12.0 g/dL; male <13.0 g/dL) or neutropenia (<1.8 x10^9/L). [3]A concurrent cytosis was defined as the presence of erythrocytosis (female, HB >16.5 g/dL or HT ≥48%; male, HB >18.5 g/dL or HT ≥52%) or leukocytosis (>10.0 x10^9/L).

	**Thrombocytopenia**	**Absence of thrombocytopenia**	**1:1 matched controls**		
	**N=631**	**N=21190**	**N=631**	**N**	**p-value**
**Age (years)**	67.0 [60.0;88.0]	65.0 [60.0;93.0]	67.0 [60.0;88.0]	22088	1.000
**Male sex**	496 (78.6%)	9252 (43.7%)	496 (78.6%)	22088	1.000
**Platelet count (10^9/L)**	137.0 [4.0;149.0]	237.0 [150.0;1196.0]	225.0 [150.0;643.0]	22088	<0.001
**Thrombocytopenia in follow-up**	217 (34.4%)	178 (0.8%)	<10 (<1.6%)	16555	<0.001
**Severe thrombocytopenia[1]**	36 (5.7%)	0 (0.0%)	0 (0.0%)	22088	<0.001
**White blood cell count (10^9/L)**	5.00 [2.10;126.60]	5.70 [2.10;111.40]	5.90 [2.80;31.40]	22087	<0.001
**Neutrophil count (10^9/L)**	2.66 [0.93;7.46]	2.98 [0.61;16.69]	3.21 [1.17;7.50]	21707	<0.001
**Basophil count (10^9/L)**	0.02 [0.00;0.11]	0.03 [0.00;0.27]	0.03 [0.00;0.19]	21707	<0.001
**Eosinophil count (10^9/L)**	0.14 [0.00;0.69]	0.16 [0.00;2.03]	0.19 [0.01;1.12]	21707	<0.001
**Lymphocyte count (10^9/L)**	1.58 [0.42;4.87]	1.89 [0.27;10.46]	1.84 [0.68;4.33]	21707	<0.001
**Monocyte count (10^9/L)**	0.44 [0.12;1.37]	0.48 [0.00;1.50]	0.53 [0.24;1.42]	21707	<0.001
**Hemoglobin concentration (g/dL)**	14.82 [10.31;18.69]	14.18 [7.09;21.11]	14.66 [8.22;18.21]	22087	0.039
**Red blood cell count (10^9/L)**	4.85 [3.25;6.36]	4.69 [3.00;8.54]	4.79 [3.52;5.96]	22087	0.186
**Hematocrit (L/L)**	0.44 [0.32;0.52]	0.43 [0.26;0.68]	0.44 [0.29;0.53]	22087	0.924
**hsCRP (mg/L)**	1.20 [0.20;48.80]	1.40 [0.10;120.00]	1.30 [0.20;35.60]	7267	0.229
**Follow-up (months)**	94.0 [5.0;136.0]	96.0 [1.0;143.0]	94.0 [2.0;137.0]	22088	0.778
**Concurrent cytopenia[2]**	97 (15.4%)	1815 (8.6%)	56 (8.9%)	21707	0.001
**Concurrent cytosis[3]**	13 (2.1%)	448 (2.2%)	16 (2.5%)	21707	0.708

N, number of evaluable individuals ≥60 years.

Supplemental Table 5 - Peripheral blood counts and baseline characteristics of the thrombocytosis cohort

A summary of baseline clinical characteristics and laboratory values for the thrombocytosis cases, 1:1 matched control cohort and all other individuals ≥60 years with peripheral blood platelet counts that are not in the cohort. Data are presented as median [min;max] for continuous variables and absolute number (%) for categorical variables. Significance was tested for thrombocytosis cases and their matched controls. A p-value below <0.05 is considered as significantly different. [1]A severe thrombocytosis was defined as platelet count >450 x10^9/L. [2]A concurrent cytopenia was defined as the presence of anemia (female <12.0 g/dL; male <13.0 g/dL) or neutropenia (<1.8 x10^9/L). [3]A concurrent cytosis was defined as the presence of erythrocytosis (female, HB >16.5 g/dL or HT ≥48%; male, HB >18.5 g/dL or HT ≥52%) or leukocytosis (>10.0 x10^9/L).

	Thrombocytosis	Absence of thrombocytosis	1:1 matched controls		
	**N=178**	**N=22096**	**N=178**	**N**	**p-value**
**Age (years)**	65.0 [60.0;87.0]	65.0 [60.0;93.0]	65.0 [60.0;87.0]	22088	1.000
**Male sex**	44 (24.7%)	10156 (46.0%)	44 (24.7%)	22088	1.000
**Platelet count (10^9/L)**	428.5 [401.0;1196.0]	234.0 [4.0;400.0]	245.5 [112.0;399.0]	22088	<0.001
**Thrombocytosis in follow-up**	71 (39.9%)	217 (1.0%)	<10 (<5.6%)	16555	<0.001
**Severe thrombocytosis[1]**	59 (33.1%)	0 (0.0%)	0 (0.0%)	22088	<0.001
**White blood cell count (10^9/L)**	7.55 [3.10;14.70]	5.70 [2.10;126.60]	5.80 [3.10;12.80]	22087	<0.001
**Neutrophil count (10^9/L)**	4.14 [1.22;10.05]	2.97 [0.61;16.69]	2.95 [1.04;8.61]	21707	<0.001
**Basophil count (10^9/L)**	0.04 [0.01;0.27]	0.03 [0.00;0.22]	0.03 [0.00;0.14]	21707	<0.001
**Eosinophil count (10^9/L)**	0.22 [0.01;1.16]	0.16 [0.00;2.03]	0.17 [0.02;0.75]	21707	<0.001
**Lymphocyte count (10^9/L)**	2.23 [0.68;4.47]	1.88 [0.27;10.46]	1.93 [0.82;4.15]	21707	0.001
**Monocyte count (10^9/L)**	0.62 [0.20;1.11]	0.48 [0.00;1.50]	0.47 [0.19;1.09]	21707	<0.001
**Hemoglobin concentration (g/dL)**	13.37 [9.18;17.08]	14.18 [7.09;21.11]	13.86 [10.31;18.21]	22087	<0.001
**Red blood cell count (10^9/L)**	4.57 [3.43;5.74]	4.70 [3.00;8.54]	4.60 [3.70;6.04]	22087	0.134
**Hematocrit (L/L)**	0.41 [0.29;0.52]	0.43 [0.26;0.68]	0.42 [0.32;0.54]	22087	<0.001
**hsCRP (mg/L)**	3.40 [0.20;59.20]	1.40 [0.10;120.00]	1.70 [0.20;26.90]	7395	0.005
**Follow-up (months)**	94.0 [6.0;135.0]	96.0 [1.0;143.0]	95.0 [5.0;136.0]	22088	0.713
**Concurrent cytopenia[2]**	27 (15.2%)	1920 (8.69%)	21 (11.8%)	21707	0.438
**Concurrent cytosis[3]**	28 (15.7%)	445 (2.01%)	<10 (<5.6%)	21707	<0.001

N, number of evaluable individuals ≥60 years.

Supplemental Table 6 – Baseline characteristics for thrombocytopenia and control cohort according to the presence of CH

A summary of blood counts and basic clinical characteristics is given for individuals with and without clonal hematopoiesis in the thrombocytopenia and 1:1 matched control cohort. Data are presented as median [min;max] for continuous variables and absolute number (%) for categorical variables. A p-value below <0.05 is considered significantly different.

	Thrombocytopenia cohort (N=612)				Control cohort (N=613)			
	No CH	CH	N	p-value	No CH	CH	N	p-value
	N=380	N=232			N=372	N=241		
**Age (years)**	65.0 [60.0;85.0]	68.0 [60.0;88.0]	612	<0.001	65.0 [60.0;83.0]	68.0 [60.0;88.0]	613	<0.001
**Male sex**	309 (81.3%)	172 (74.1%)	612	0.042	293 (78.8%)	188 (78.0%)	613	0.841
**Platelet count (10^9/L)**	137.0 [4.0;149.0]	135.5 [19.0;149.0]	612	0.475	224.5 [155.0;401.0]	226.0 [150.0;643.0]	613	0.498
**Thrombocytopenia at follow-up**	136 (35.8%)	75 (32.3%)	458	0.184	<10	<10	465	0.010
**White blood cell count (10^9/L)**	5.10 [2.60;59.50]	5.00 [2.40;25.90]	611	0.073	5.80 [2.90;31.40]	6.00 [2.80;10.50]	613	0.180
**Neutrophil count (10^9/L)**	2.71 [0.93;6.03]	2.64 [1.03;7.46]	584	0.514	3.07 [1.39;7.39]	3.41 [1.17;7.50]	598	0.021
**Basophil count (10^9/L)**	0.02 [0.00;0.10]	0.02 [0.00;0.11]	584	0.044	0.03 [0.00;0.19]	0.03 [0.00;0.10]	598	0.473
**Eosinophil count (10^9/L)**	0.14 [0.01;0.69]	0.13 [0.00;0.66]	584	0.008	0.19 [0.01;1.12]	0.19 [0.02;0.96]	598	0.640
**Lymphocyte count (10^9/L)**	1.62 [0.42;3.79]	1.52 [0.60;4.87]	584	0.093	1.85 [0.90;4.33]	1.82 [0.68;4.23]	598	0.146
**Monocyte count (10^9/L)**	0.44 [0.14;1.03]	0.43 [0.12;1.37]	584	0.252	0.51 [0.24;1.11]	0.54 [0.24;1.42]	598	0.184
**Hemoglobin concentration (g/dL)**	14.82 [10.47;17.72]	14.66 [10.31;18.69]	611	0.017	14.66 [8.22;18.21]	14.66 [11.76;18.05]	613	0.876
**Red blood cell count (10^9/L)**	4.85 [3.34;6.36]	4.83 [3.25;5.89]	611	0.010	4.78 [3.77;5.96]	4.80 [3.52;5.88]	613	0.644
**Hematocrit (L/L)**	0.44 [0.33;0.52]	0.44 [0.32;0.52]	611	0.021	0.44 [0.29;0.53]	0.44 [0.34;0.53]	613	0.545
**hsCRP (mg/L)**	1.20 [0.20;48.80]	1.10 [0.20;15.10]	222	0.238	1.30 [0.20;35.60]	1.30 [0.20;13.00]	191	0.412
**Concurrent anemia**	<10	22 (9.5%)	611	<0.001	17 (4.6%)	11 (4.6%)	613	1.000
**Concurrent neutropenia**	39 (10.3%)	28 (12.1%)	584	0.506	16 (4.3%)	13 (5.4%)	598	0.563
**Concurrent cytosis**	<10	<10	584	0.755	<10	<10	598	0.797

N, number of individuals.

Supplemental Table 7 – Baseline characteristics for thrombocytosis and control cohort according to the presence of CH

A summary of blood counts and basic clinical characteristics is given for individuals with and without clonal hematopoiesis in the thrombocytosis and 1:1 matched control cohort. Data are presented as median [min;max] for continuous variables and absolute number (%) for categorical variables. A p-value below <0.05 is considered significantly different.

	Thrombocytosis cohort (N=172)				Control cohort (n=175)			
	No CH	CH	N	p-value	No CH	CH	N	p-value
	N=76	N=96			N=109	N=66		
**Age (years)**	64.0 [60.0;81.0]	66.0 [60.0;87.0]	172	0.018	65.0 [60.0;87.0]	65.5 [60.0;84.0]	175	0.076
**Male sex**	16 (21.1%)	27 (28.1%)	172	0.376	25 (22.9%)	17 (25.8%)	175	0.717
**Platelet count (10^9/L)**	422.0 [401.0;646.0]	434.5 [401.0;1196.0]	172	0.004	244.0 [112.0;391.0]	247.0 [140.0;399.0]	175	0.880
**Thrombocytosis at follow-up**	31 (40.8%)	38 (39.6%)	112	0.532	<10	<10	125	0.362
**White blood cell count (10^9/L)**	7.35 [4.30;14.70]	7.55 [4.00;13.70]	172	0.617	5.40 [3.10;12.80]	6.20 [3.30;9.80]	175	0.154
**Neutrophil count (10^9/L)**	3.97 [1.79;9.43]	4.30 [1.22;10.05]	163	0.169	2.84 [1.12;8.61]	3.18 [1.04;6.02]	170	0.116
**Basophil count (10^9/L)**	0.04 [0.01;0.10]	0.05 [0.01;0.27]	163	0.042	0.03 [0.00;0.12]	0.03 [0.01;0.14]	170	0.790
**Eosinophil count (10^9/L)**	0.21 [0.01;1.16]	0.22 [0.08;0.89]	163	0.187	0.17 [0.03;0.75]	0.16 [0.02;0.68]	170	0.525
**Lymphocyte count (10^9/L)**	2.24 [1.27;4.47]	2.21 [1.09;3.65]	163	0.215	1.97 [0.99;4.15]	1.91 [0.82;3.56]	170	1.000
**Monocyte count (10^9/L)**	0.62 [0.31;1.11]	0.62 [0.28;1.09]	163	0.726	0.45 [0.20;1.03]	0.49 [0.19;1.09]	170	0.211
**Hemoglobin concentration (g/dL)**	13.21 [10.96;15.63]	13.45 [9.18;17.08]	172	0.469	13.86 [10.96;16.76]	13.86 [10.31;18.21]	175	0.456
**Red blood cell count (10^9/L)**	4.57 [3.56;5.28]	4.59 [3.43;5.74]	172	0.995	4.59 [3.70;6.04]	4.62 [3.79;5.90]	175	0.621
**Hematocrit (L/L)**	0.41 [0.33;0.47]	0.41 [0.29;0.52]	172	0.764	0.42 [0.35;0.51]	0.43 [0.32;0.54]	175	0.511
**hsCRP (mg/L)**	4.80 [0.40;36.70]	3.00 [0.20;59.20]	58	0.409	1.90 [0.20;9.70]	1.10 [0.30;26.90]	65	0.514
**Concurrent anemia**	<10	18 (18.8%)	172	0.086	<10	<10	175	0.428
**Concurrent leukocytosis**	11 (14.5%)	13 (13.5%)	172	1.000	<10	0 (0.0%)	175	0.528
**Concurrent cytopenia**	<10	18 (18.8%)	163	0.198	12 (11.0%)	<10	170	0.636
**Concurrent cytosis**	11 (14.5%)	15 (15.6%)	163	1.000	<10	<10	170	0.633

N, number of individuals.

Supplemental Table 8 – Univariable and multivariable associations of clonal hematopoiesis with overall survival in individuals with platelet count abnormalities

Results are shown for the thrombocytopenia (A) and thrombocytosis (B) cases and the respective matched controls. The absence of clonal hematopoiesis is used as a reference.

A

	N	HR (95% CI) Univariable analysis	P	HR (95% CI) Multivariable analysis*	P
**Thrombocytopenia cohort**					
**No CH**	380	1		1	
**CH**	232	1.77 (1.17-2.67)	0.007	1.32 (0.85-2.03)	0.215
**Control cohort**					
**No CH**	372	1		1	
**CH**	241	1.05 (0.66-1.69)	0.825	0.78 (0.48-1.28)	0.327

*Age and sex used as covariates

CH, clonal hematopoiesis; HR, hazard ratio; CI, confidence interval; N, number of individuals

B

	N	HR (95% CI) Univariable analysis	P	HR (95% CI) Multivariable analysis*	P
**Thrombocytosis cohort**					
**No CH**	76	1		1	
**CH**	96	0.87 (0.35-2.15)	0.767	0.52 (0.19-1.42)	0.202
**Control cohort**					
**No CH**	109	1		1	
**CH**	66	1.25 (0.43-3.59)	0.684	0.87 (0.28-2.68)	0.809

*Age and sex used as covariates

CH, clonal hematopoiesis; HR, hazard ratio; CI, confidence interval; N, number of individuals

Supplemental Table 9 – Univariable and multivariable associations for the number of mutated genes with overall survival in individuals with platelet count abnormalities

Results are shown for the thrombocytopenia (A) and thrombocytosis (B) cases and the matched controls. The cohort of individuals without CH or the presence of 1 mutated gene is used as reference.

A

	N	HR (95% CI) Univariable analysis	P	HR (95% CI) Multivariable analysis*	P
**Thrombocytopenia cohort**					
**Absence of CH or 1 mutated gene**	555			1	
**≥2 mutated genes**	57	2.98 (1.80-4.95)	<0.001	2.08 (1.24-3.50)	0.006
**Control cohort**					
**Absence of CH or 1 mutated gene**	583	1		1	
**≥2 mutated genes**	30	1.54 (0.62-3.83)	0.352	0.98 (0.39-2.47)	0.967

*Age and sex used as covariates

HR, hazard ratio; CI, confidence interval; N, number of individuals

B

	N	HR (95% CI) Univariable analysis	P	HR (95% CI) Multivariable analysis*	P
**Thrombocytosis cohort**					
**Absence of CH or 1 mutated gene**	145	1		1	
**≥2 mutated genes**	27	1.54 (0.51–4.65)	0.446	0.67 (0.20-2.27)	0.521
**Control cohort**					
**Absence of CH or 1 mutated gene**	163	1		1	
**≥2 mutated genes**	12	0.00 (0.00-Inf)	0.997	0.00 (0.00-Inf)	0.998

*Age and sex used as covariates

HR, hazard ratio; CI, confidence interval; N, number of individuals

Supplemental Table 10 – Univariable and multivariable associations of clone size with overall survival for individuals with platelet count abnormalities

Results are shown for the thrombocytopenia (A) and thrombocytosis (B) cases and the matched controls. The cohort of individuals without CH or a VAF of <5% is used as reference in all analyses.

A

	N	HR (95% CI) Univariable analysis	P	HR (95% CI) Multivariable analysis*	P
**Thrombocytopenia cohort**					
**Absence of CH or VAF <5%**	524	1		1	
**VAF ≥5%**	88	2.11 (1.30-3.41)	0.002	1.56 (0.95-2.55)	0.079
**Control cohort**					
**Absence of CH or VAF <5%**	547	1		1	
**VAF ≥5%**	66	0.89 (0.41-1.95)	0.775	0.79 (0.36-1.72)	0.552

*Age and sex used as covariates

HR, hazard ratio; CI, confidence interval; N, number of individuals

B

	N	HR (95% CI) Univariable analysis	P	HR (95% CI) Multivariable analysis*	P
**Thrombocytosis cohort**					
**Absence of CH or VAF <5%**	130	1		1	
**VAF ≥5%**	42	0.86 (0.28–2.58)	0.783	0.52 (0.16-1.68)	0.278
**Control cohort**					
**Absence of CH or VAF <5%**	153	1		1	
**VAF ≥5%**	22	0.51 (0.07-3.93)	0.521	0.47 (0.06-3.63)	0.471

*Age and sex used as covariates

HR, hazard ratio; CI, confidence interval; N, number of individuals

Supplemental Table 11 – Univariable and multivariable associations of specific gene mutations with overall survival for individuals with platelet count abnormalities

Results are shown for the thrombocytopenia (A) and thrombocytosis (B) cases. The absence of a mutation in the respective gene is used as a reference. Analyses were restricted to genes with ≥5 mutated individuals.

A

Gene	Events/N	Univariable HR (95% CI)	P	Multivariable* HR (95% CI)	P
**DNMT3A**	23/146	1.14 (0.71-1.83)	0.592	0.94 (0.58-1.51)	0.787
**TET2**	19/77	1.90 (1.15-3.16)	0.013	1.23 (0.72-2.09)	0.447
**ASXL1**	3/19	1.10 (0.35-3.47)	0.873	0.78 (0.24-2.50)	0.677
**Spliceosome**	8/21	3.53 (1.71-7.32)	<0.001	2.69 (1.29-5.63)	0.009
**TP53**	6/11	5.81 (2.53-13.34)	<0.001	5.83 (2.49-13.64)	<0.001

*Age and sex used as covariates

HR, hazard ratio; CI, confidence interval; N, number of individuals

B

Gene	Events/N	Univariable HR (95% CI)	P	Multivariable* HR (95% CI)	P
**DNMT3A**	1/45	0.15 (0.02-1.12)	0.065	0.14 (0.02–1.06)	0.057
**TET2**	4/27	1.52 (0.50-4.58)	0.458	1.03 (0.33-3.20)	0.954
**JAK2**	4/22	1.92 (0.63-5.78)	0.249	1.30 (0.41-4.06)	0.654
**ASXL1**	3/9	3.47 (1.00-11.96)	0.049	2.52 (0.68-9.32)	0.166
**CALR**	1/8	1.11 (0.15-8.29)	0.921	0.47 (0.06-3.78)	0.477
**Spliceosome**	1/7	1.23 (0.16-9.19)	0.843	0.82 (0.11-6.29)	0.845

*Age and sex used as covariates

HR, hazard ratio; CI, confidence interval; N, number of individuals

Supplemental Table 12 – Univariable and multivariable associations of clonal hematopoiesis with overall survival in isolated thrombocytopenia/thrombocytosis or with a concurrent cytopenia/cytosis

Results are shown for the thrombocytopenia (A) and thrombocytosis (B) cases and the respective matched controls. The absence of clonal hematopoiesis is used as a reference.

A

	N	HR (95% CI) Univariable analysis	P	HR (95% CI) Multivariable analysis*	P
**Isolated thrombocytopenia**					
**No CH**	335	1		1	
**CH**	184	1.51 (0.93-2.43)	0.092	1.16 (0.70-1.90)	0.570
**Thrombocytopenia with a concurrent cytopenia**					
**No CH**	45	1		1	
**CH**	48	2.61 (1.01-6.74)	0.047	1.56 (0.52-4.68)	0.430

*Age and sex used as covariates

CH, clonal hematopoiesis; HR, hazard ratio; CI, confidence interval; N, number of individuals

B

	N	HR (95% CI) Univariable analysis	P	HR (95% CI) Multivariable analysis*	P
**Isolated thrombocytosis**					
**No CH**	65	1		1	
**CH**	81	0.79 (0.30-2.11)	0.638	0.52 (0.18-1.50)	0.227
**Thrombocytosis with a concurrent cytosis**					
**No CH**	11	1		1	
**CH**	15	1.41 (0.13-15.54)	0.780	0.36 (0.01-10.82)	0.554

*Age and sex used as covariates

CH, clonal hematopoiesis; HR, hazard ratio; CI, confidence interval; N, number of individuals

Supplemental Table 13 – Incident diagnoses of hematological malignancies

Data on incident malignancies was obtained by linkage to the Netherlands Cancer Registry (NCR). The total number of evaluable individuals after linkage as well as number of incident malignancies are shown for the thrombocytopenia (A) and thrombocytosis (B) cases and respective matched controls.

A

	**Total number of evaluable individuals**	**Hematological malignancies**
**Thrombocytopenia with CH**	223	10
**Thrombocytopenia without CH**	372	9
**Controls with CH**	241	6
**Controls without CH**	369	5

CH, clonal hematopoiesis

B

	**Total number of evaluable individuals**	**Hematological malignancies**
**Thrombocytosis with CH**	93	21
**Thrombocytosis without CH**	76	<5
**Controls with CH**	65	<5
**Controls without CH**	108	<5

CH, clonal hematopoiesis

Supplemental Figure 1 – Coverage in thrombocytopenia cohort

Number of aligned consensus reads (A) and the raw number of aligned reads (B) for all genes in our sequencing panel for the thrombocytopenia cohort (n=1225). Columns and error bars indicate median and interquartile range respectively.

A

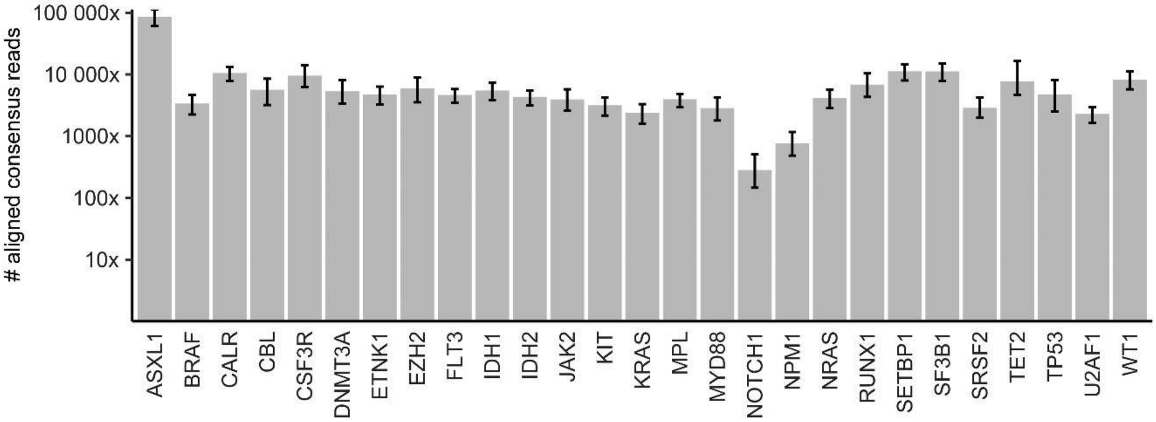

B

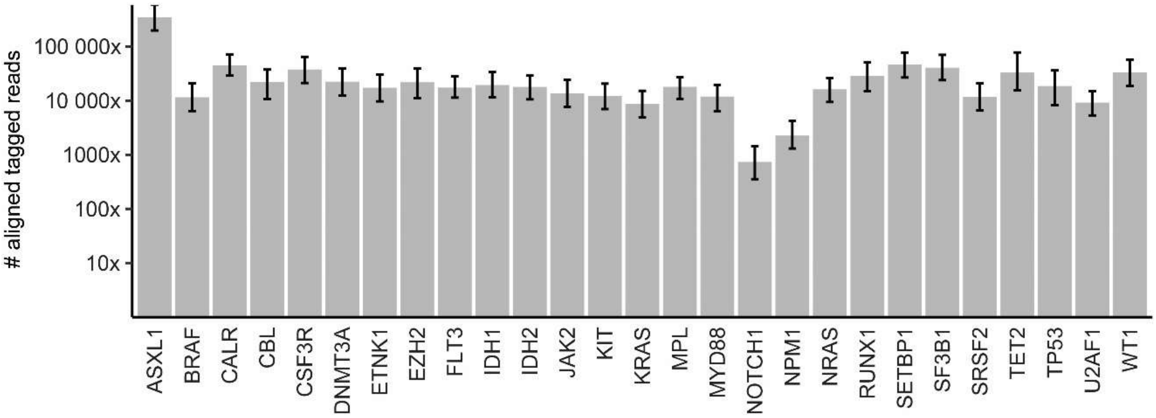

Supplemental Figure 2 – Coverage in thrombocytosis cohort

Number of aligned consensus reads (A) and the raw number of aligned reads (B) for all genes in our sequencing panel for the thrombocytosis cohort (n=347). Columns and error bars indicate median and interquartile range respectively.

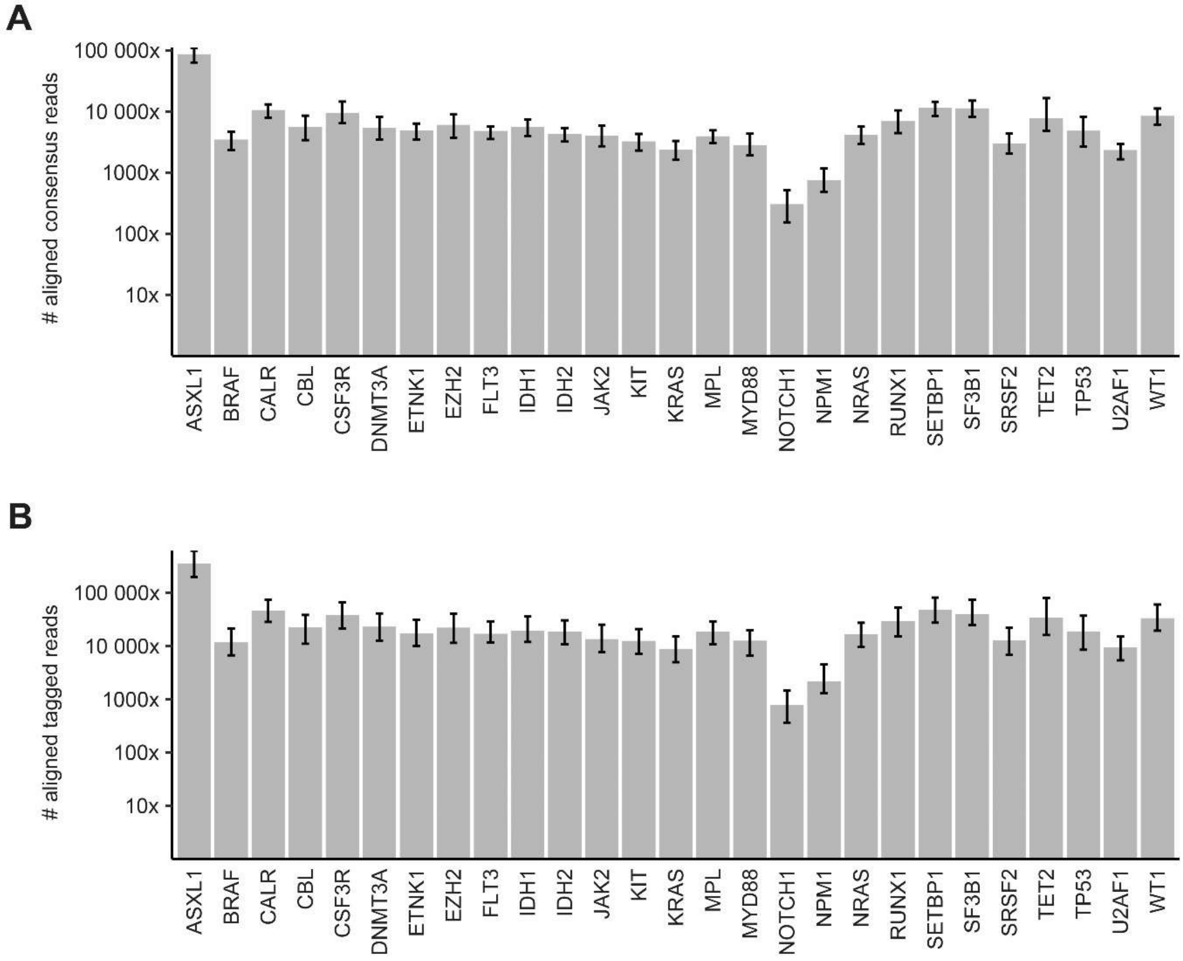

Supplemental Figure 3 – Prevalence of thrombocytosis in the population-based Lifelines cohort ≥60 years

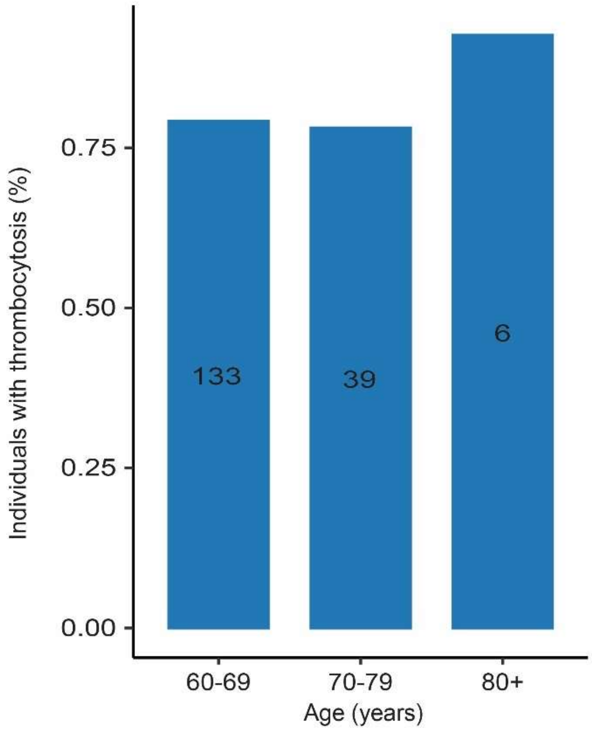

Supplemental Figure 4 –Mutational spectrum for mild and severe thrombocytopenia or thrombocytosis

Individuals with mild or severe thrombocytopenia or thrombocytosis compared with their respective 1:1 matched controls. (A-B) Percentage of CH in individuals with mild or severe thrombocytopenia (light blue) and mild or severe thrombocytosis (dark blue) compared to respective matched controls (grey). (C-D) The number of mutated genes in individuals with mild or severe thrombocytopenia (light blue) and mild or severe thrombocytosis (dark blue) compared to respective matched controls (grey). (E-F) The highest VAF (%) in individuals with mild or severe thrombocytopenia (light blue) and mild or severe thrombocytosis (dark blue) compared to respective matched controls (grey). Only individuals with CH are displayed in these figures.

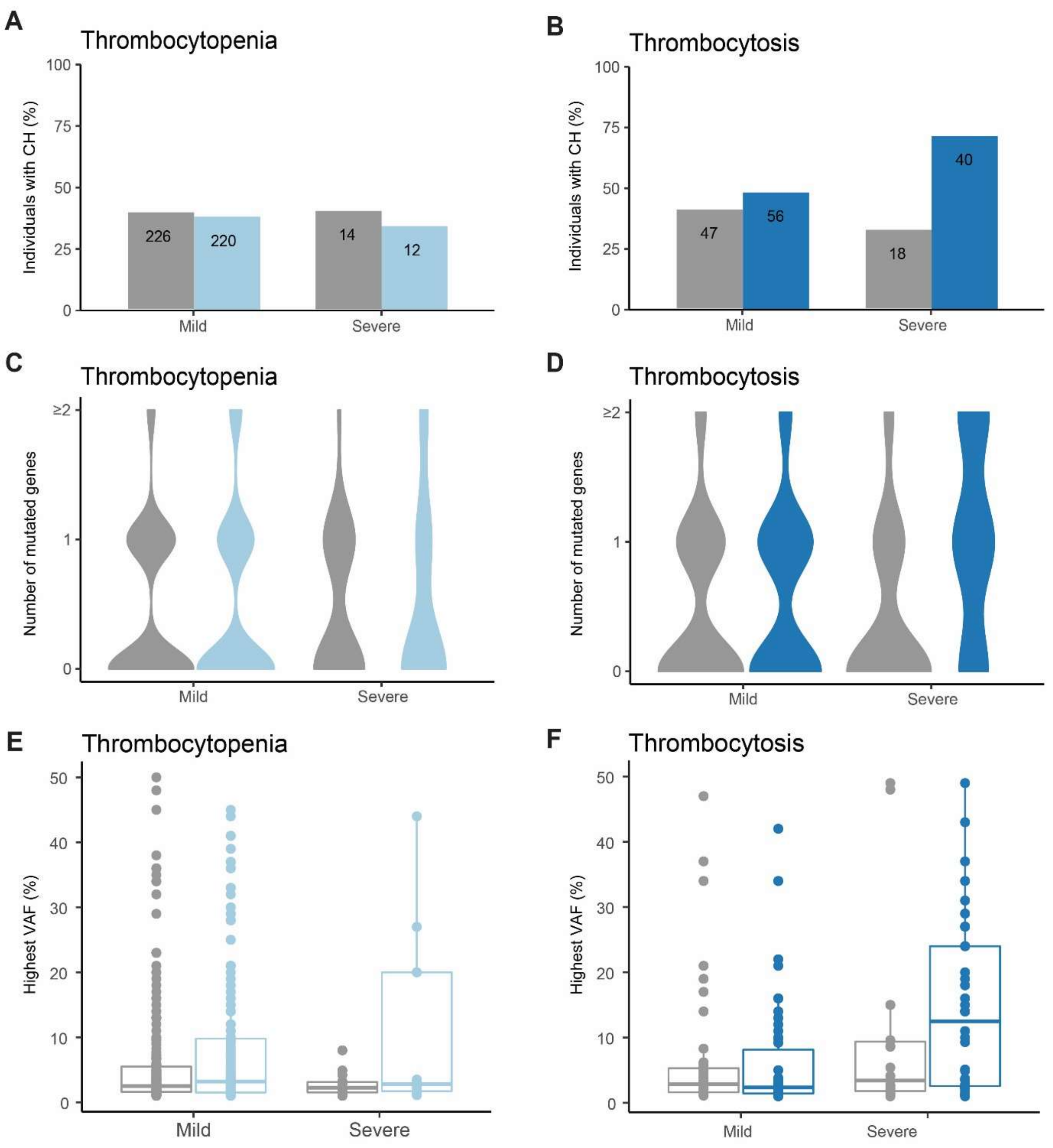

Supplemental Figure 5 – Mutational landscapes for mild and severe thrombocytopenia

(A) Mutational landscape for individuals with mild thrombocytopenia and CH defined as platelet counts 100-150x10^9/L (n=220). (B) Mutational landscape for the 1:1 matched controls for individuals with mild thrombocytopenia (n=226). (C) Mutational landscape for individuals with severe thrombocytopenia and CH defined as platelet counts <100x10^9/L (n=12). (D) Mutational landscape for the 1:1 matched controls for individuals with severe thrombocytopenia (n=14).

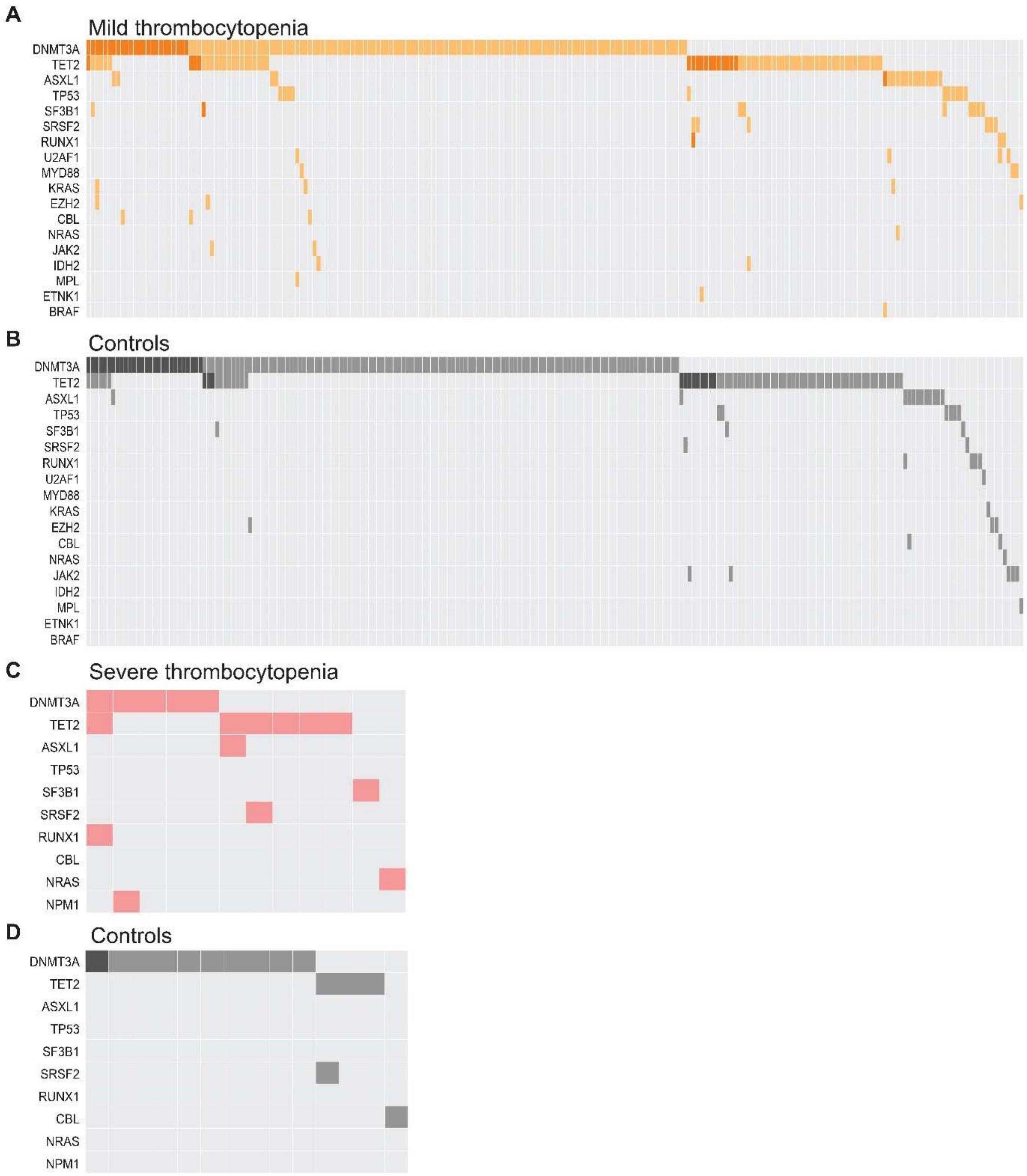

Supplemental Figure 6 – Mutational landscapes for mild and severe thrombocytosis

(A) Mutational landscape for individuals with mild thrombocytosis and CH defined as platelet counts 400-450x10^9/L (n=56). (B) Mutational landscape for the 1:1 matched controls for cases with mild thrombocytosis (n=47). (C) Mutational landscape for individuals with severe thrombocytosis and CH defined as platelet counts >450x10^9/L (n=40). (D) Mutational landscape for the 1:1 matched controls for cases with severe thrombocytosis (n=18).

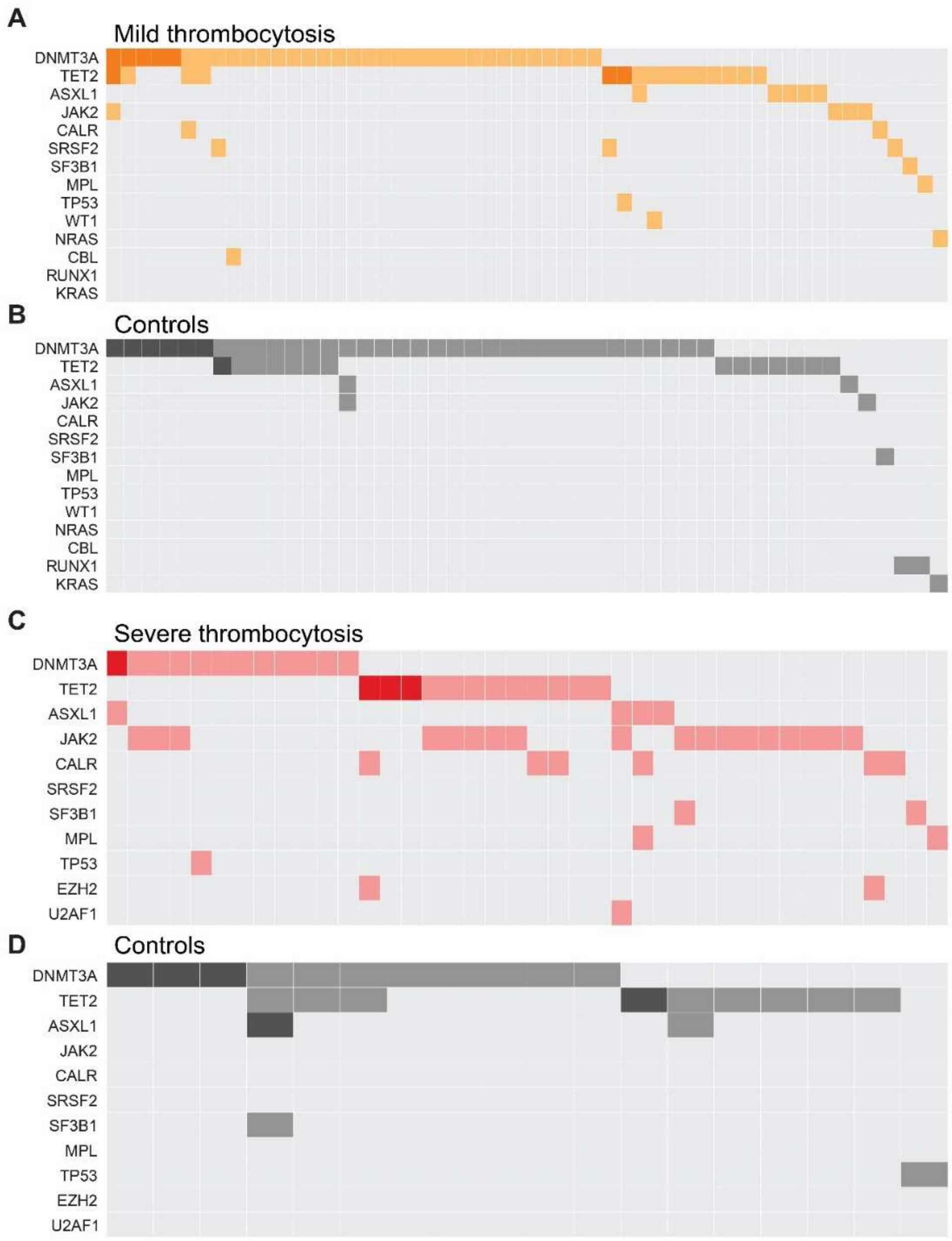

Supplemental Figure 7 – Co-mutational patterns in individuals with platelet count abnormalities and their matched controls

(A) Chord diagram showing co-mutational patterns in thrombocytopenia cases. The width of the ribbon corresponds to the relative frequency of the co-occurrence, whereas the length of the arc corresponds to the relative frequency of mutations. (B) Chord diagram showing co-mutational patterns in 1:1 matched controls for the thrombocytopenia cases. (C) Chord diagram showing co-mutational patterns in thrombocytosis cases. (D) Chord diagram showing co-mutational patterns in 1:1 matched controls for the thrombocytosis cases. Spliceosome mutations include *SF3B1*, *SRSF2* and *U2AF1*.

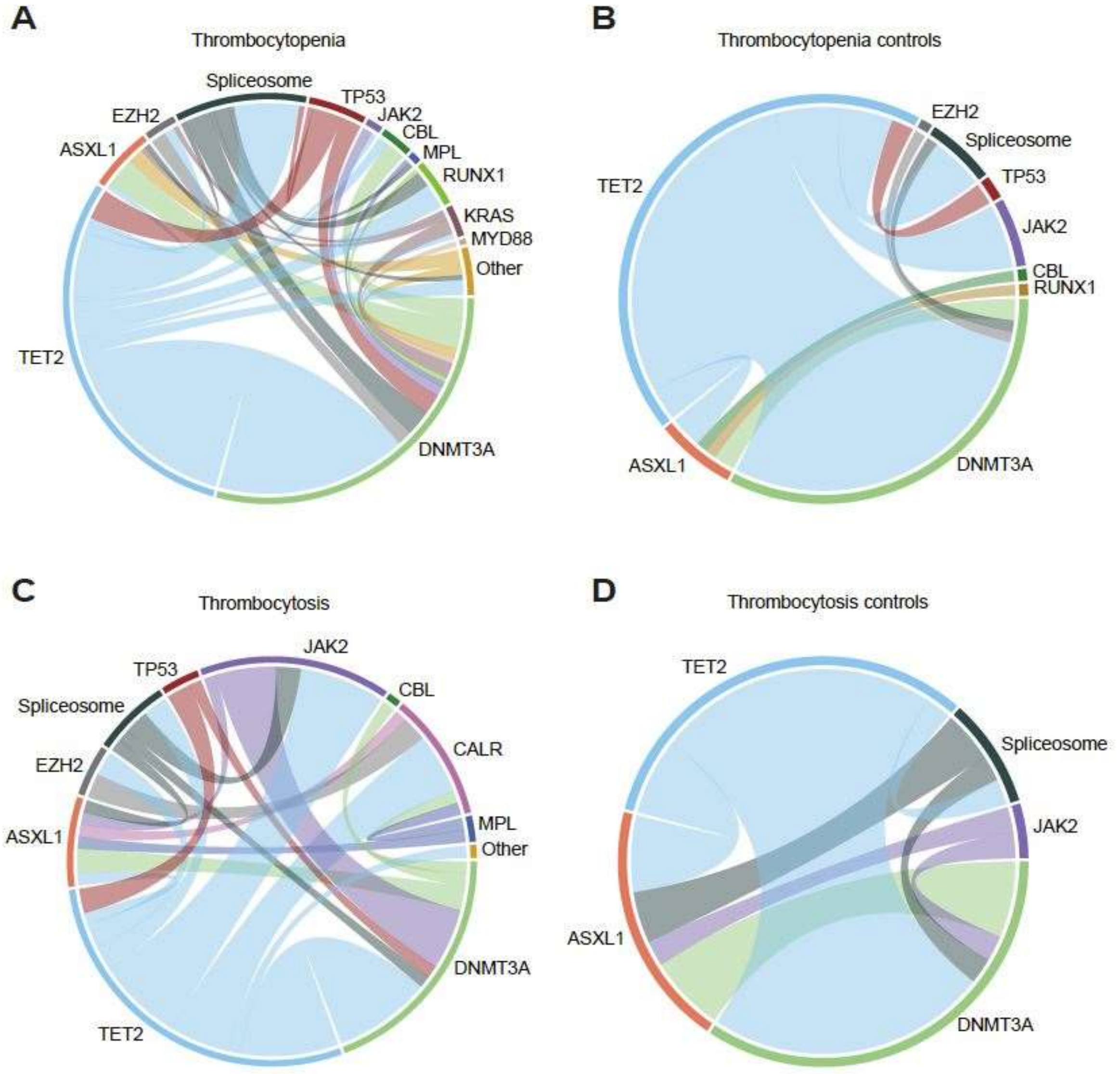

Supplemental Figure 8 – Pyramid plots of all detected gene mutations

(A) Pyramid plot indicating the proportion of individuals with hematological driver gene mutations for individuals with thrombocytopenia (light blue), and 1:1 matched controls (grey). Mutations involved in the spliceosome are categorized in one group containing the genes *SF3B1*, *SRSF2* and *U2AF1*. (B) Pyramid plot indicating the proportion of individuals with hematological driver gene mutations for individuals with thrombocytosis (dark blue), and 1:1 matched controls (grey).

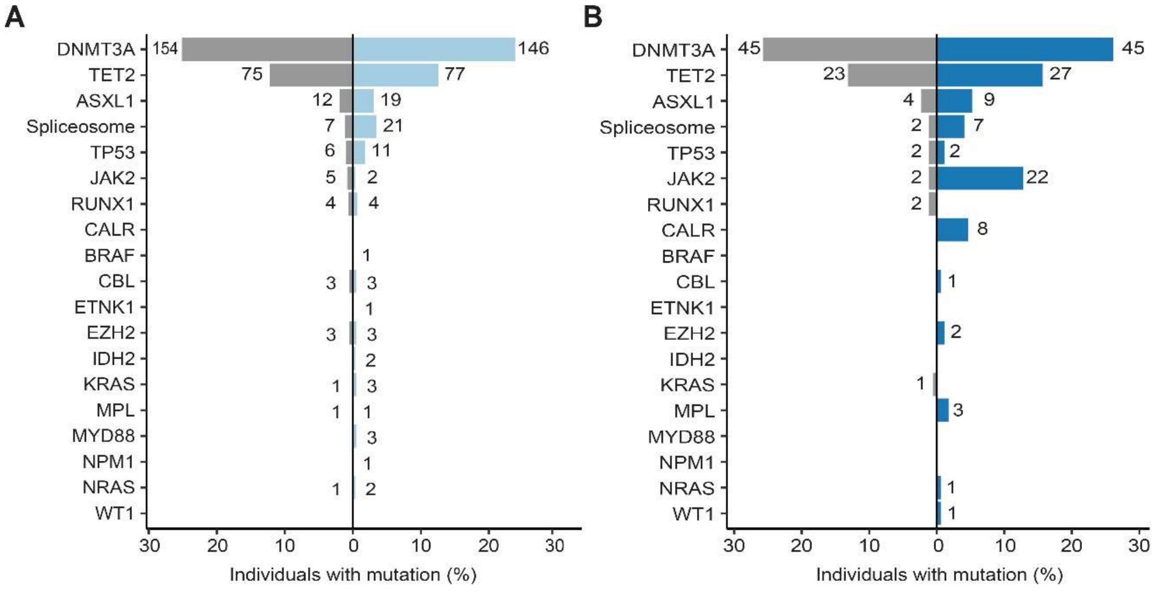

Supplemental Figure 9 – Variant allele frequency distribution per gene

(A) Variant allele frequencies for all detected somatic mutations in genes with at least 5 mutations in individuals with thrombocytopenia (light blue), and the 1:1 matched controls (grey). (B) Variant allele frequencies for all detected somatic mutations in genes with at least 5 mutations in individuals with thrombocytosis (dark blue), and the 1:1 matched controls (grey). A significant difference was observed between thrombocytosis cases compared to their matched controls for the VAF of *DNMT3A* (P=0.018).

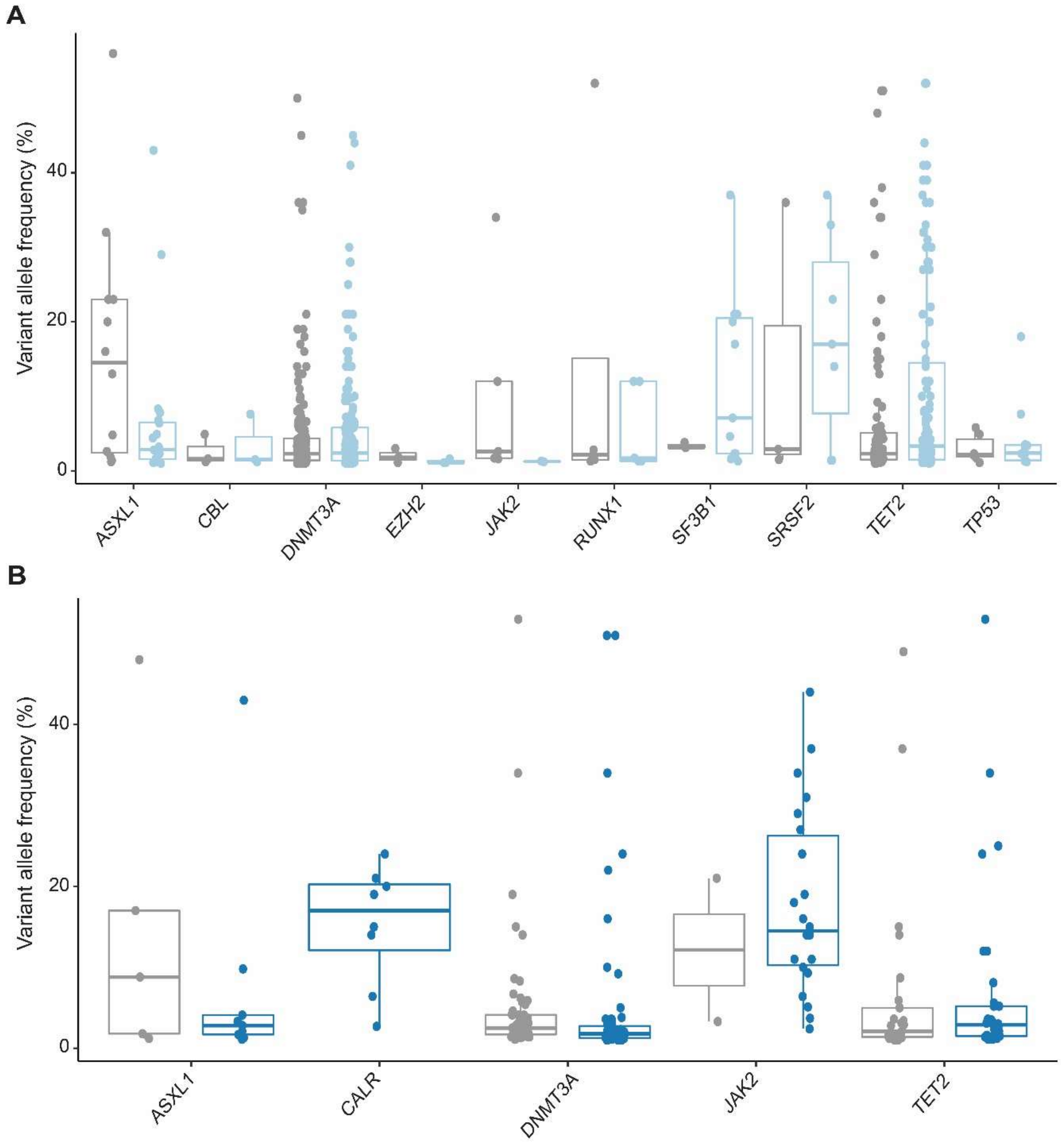

Supplemental Figure 10 – Relation between VAF of commonly mutated genes and absolute platelet count for individuals with thrombocytopenia

(A) Relation between absolute platelet count (x10^9/L) and VAF (%) at baseline for individuals with thrombocytopenia. (B) Relation between absolute platelet count (x10^9/L) and DNMT3A VAF (%) at baseline for individuals with thrombocytopenia. (C) Relation between absolute platelet count (x10^9/L) and TET2 VAF (%) at baseline for individuals with thrombocytopenia. (D) Relation between absolute platelet count (x10^9/L) and ASXL1 VAF (%) at baseline for individuals with thrombocytopenia. (E) Relation between absolute platelet count (x10^9/L) and TP53 VAF (%) at baseline for individuals with thrombocytopenia.

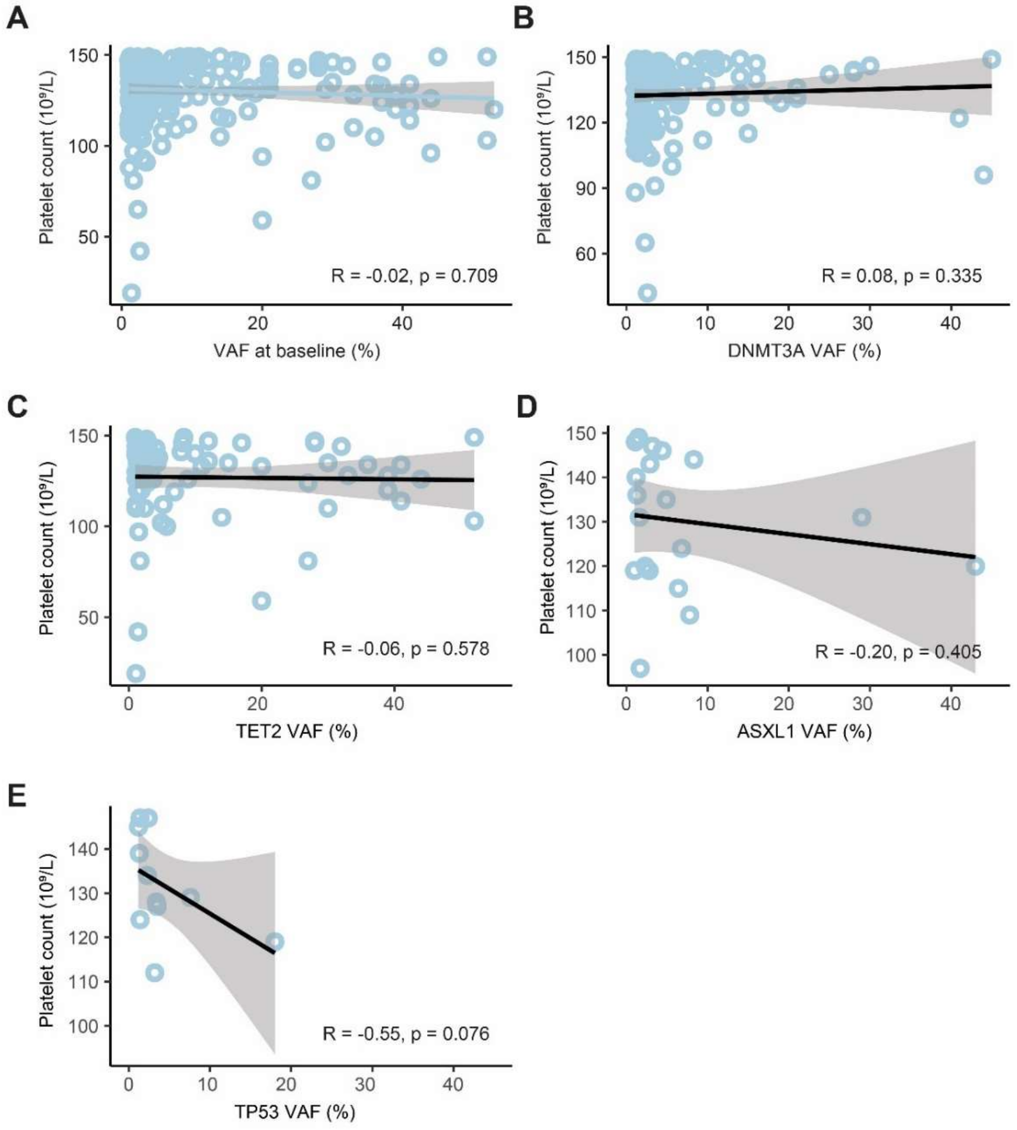

Supplemental Figure 11 - Relation between VAF of commonly mutated genes and absolute platelet count for individuals with thrombocytosis

(A) Relation between absolute platelet count (x10^9/L) and VAF (%) at baseline for individuals with thrombocytosis. (B) Relation between absolute platelet count (x10^9/L) and DNMT3A VAF (%) at baseline for individuals with thrombocytosis. (C) Relation between absolute platelet count (x10^9/L) and TET2 VAF (%) at baseline for individuals with thrombocytosis. (D) Relation between absolute platelet count (x10^9/L) and ASXL1 VAF (%) at baseline for individuals with thrombocytosis.

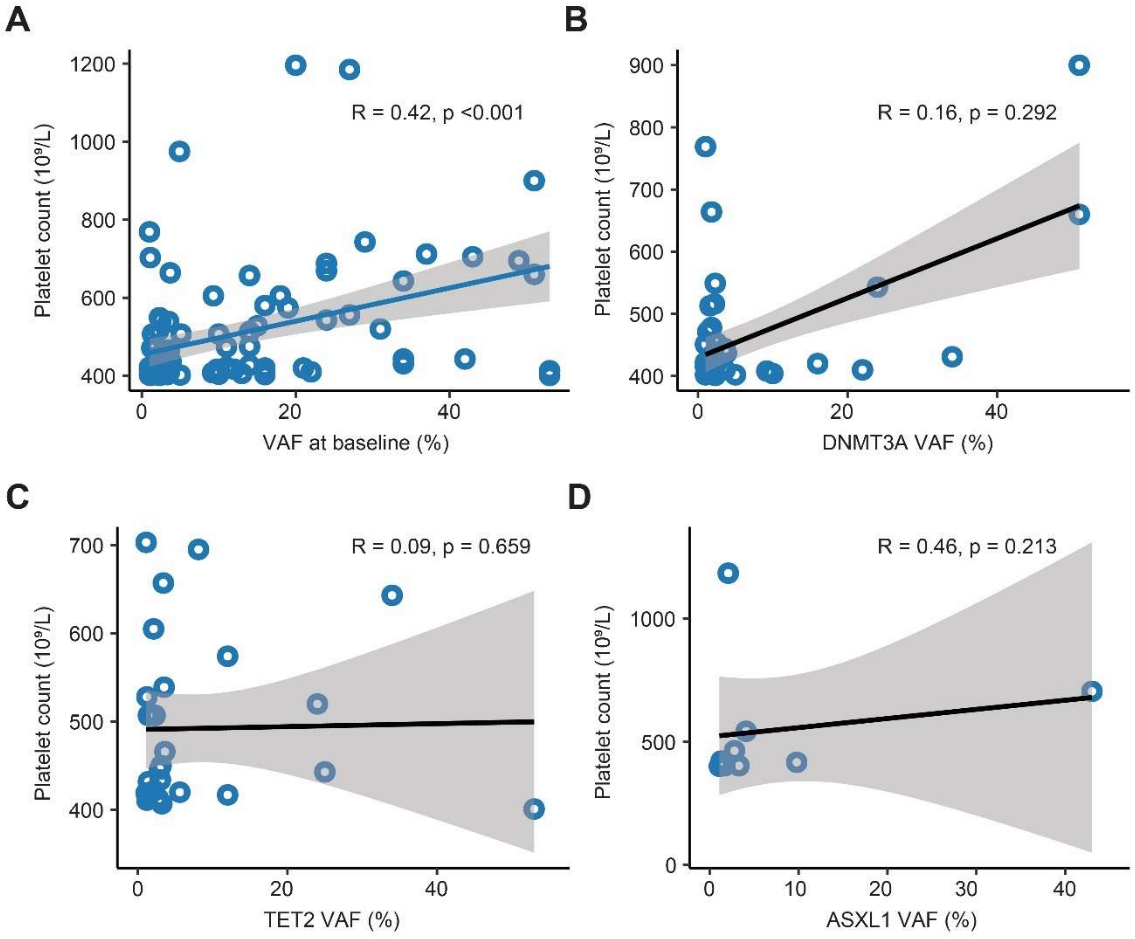

Supplemental Figure 12 – Mutational spectrum for isolated thrombocytopenia/thrombocytosis or with concurrent cytopenia/cytosis

For all analyses, subgroups of thrombocytopenia/thrombocytosis cases are compared with the respective 1:1 matched controls. (A-B) Number of mutated genes in individuals with isolated thrombocytopenia (light blue) or isolated thrombocytosis (dark blue) versus cases with a concurrent cytopenia (light blue) or a concurrent cytosis (dark blue) and matched controls (grey). (C-D) Highest detected VAF (%) in individuals with isolated thrombocytopenia (light blue) or isolated thrombocytosis (dark blue) versus cases with a concurrent cytopenia (light blue) or a concurrent cytosis (dark blue) and matched controls (grey). Only individuals with CH are displayed in these figures. (E-F) Pyramid plot indicating the proportion of individuals with specific gene mutations for individuals with isolated thrombocytopenia (light blue) or isolated thrombocytosis (dark blue) versus cases with a concurrent cytopenia (light green) or concurrent cytosis (dark green) compared to respective matched controls (grey). Spliceosome mutations include *SF3B1*, *SRSF2* and *U2AF1*.

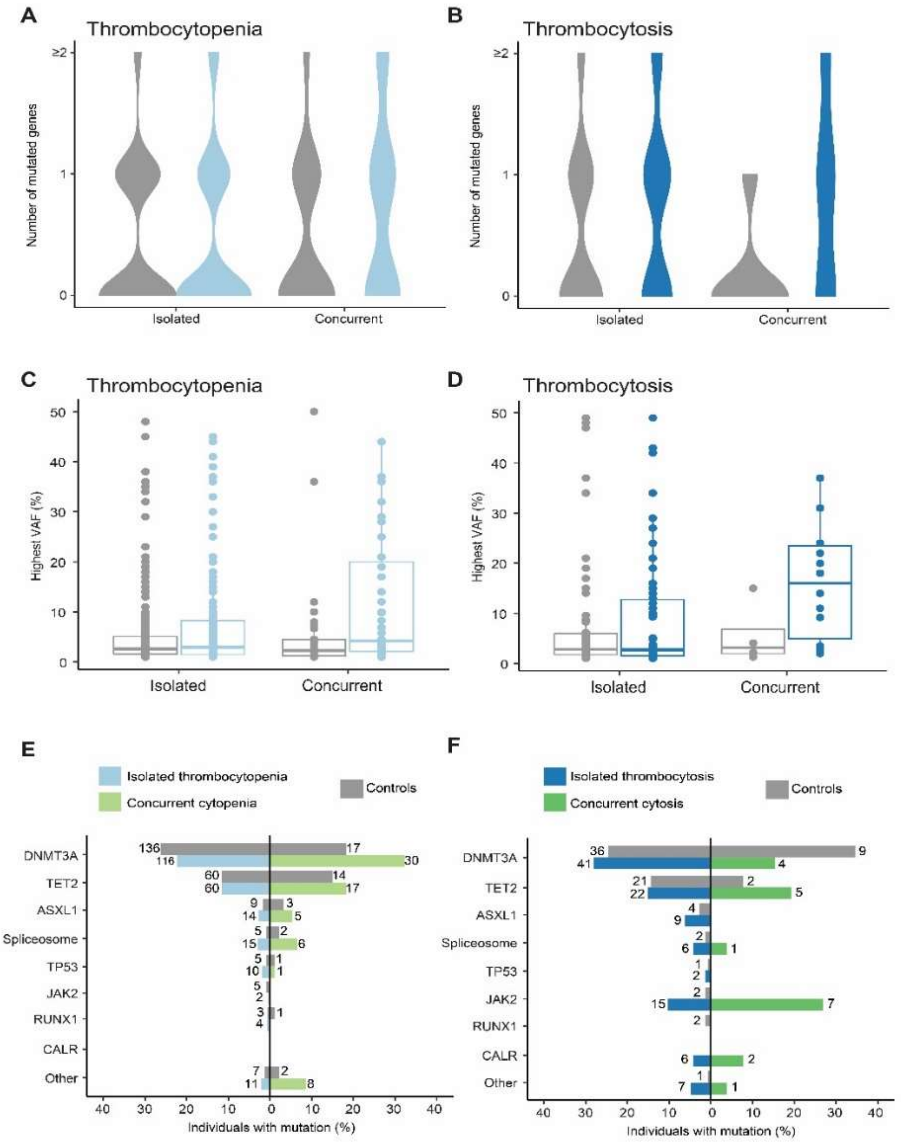

Supplemental Figure 13 – Mutational landscapes for thrombocytopenia with concurrent cytopenia and thrombocytosis with concurrent cytosis

(A) Mutational landscape for individuals with thrombocytopenia and anemia (n=22). (B) Mutational landscape for individuals with thrombocytopenia and neutropenia (n=28). (C) Mutational landscape for individuals with thrombocytosis and leukocytosis (n=13). Concurrent cytopenia included anemia (female <12.0 g/dL HB; male <13.0 g/dL HB) or neutropenia (neutrophil count <1.8 x10^9/L), concurrent cytosis was defined as erythrocytosis (female, HB >16.5 g/dL or HT ≥48%; male, HB >18.5 g/dL or HT ≥52%) or leukocytosis (white blood cell count >10.0 x10^9/L). There were 2 individuals with thrombocytosis and erythrocytosis and both carried a mutation in JAK2.

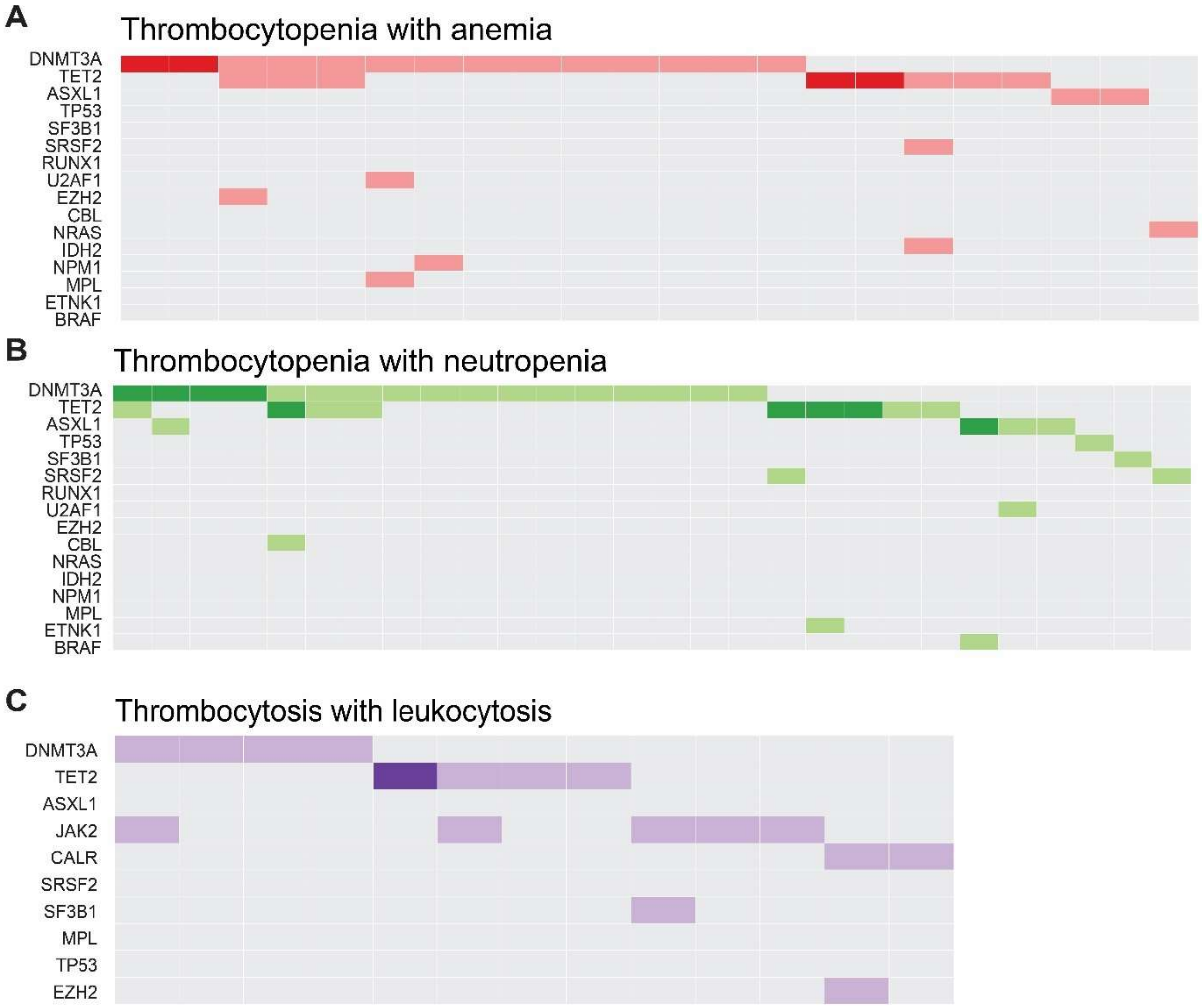

Supplemental Figure 14 – Evolution of platelet counts over time

(A) Change in platelet counts ($x10^9$) over time for individuals with stable and corrected thrombocytopenia and the individuals without follow-up data. (B) Change in platelet counts ($x10^9$) over time for individuals with stable and corrected thrombocytosis and the individuals without follow-up data. The median follow-up period was 94 months (range 5-136 months). The dashed line indicates the clinical cut-off value for thrombocytopenia (lower dashed line in both figures) and thrombocytosis (upper dashed line in both figures).

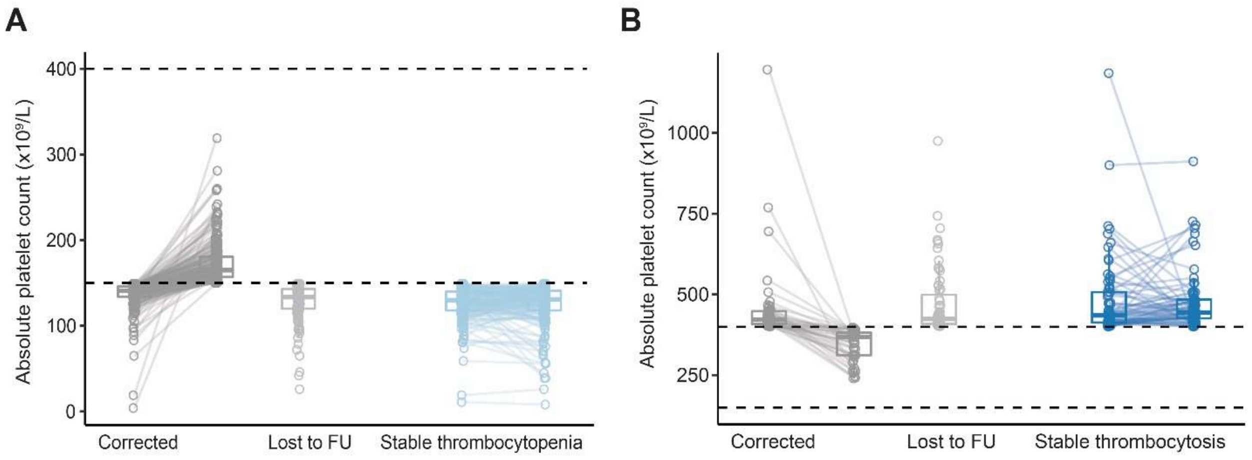

Supplemental Figure 15 - Mutational landscapes for persistent and corrected thrombocytopenia and thrombocytosis

(A) Mutational landscape for individuals with persistent thrombocytopenia and CH (n=75). (B) Mutational landscape for individuals with corrected thrombocytopenia and CH (n=89). (C) Mutational landscape for individuals with persistent thrombocytosis and CH (n=38). (D) Mutational landscape for individuals with corrected thrombocytosis and CH (n=27).

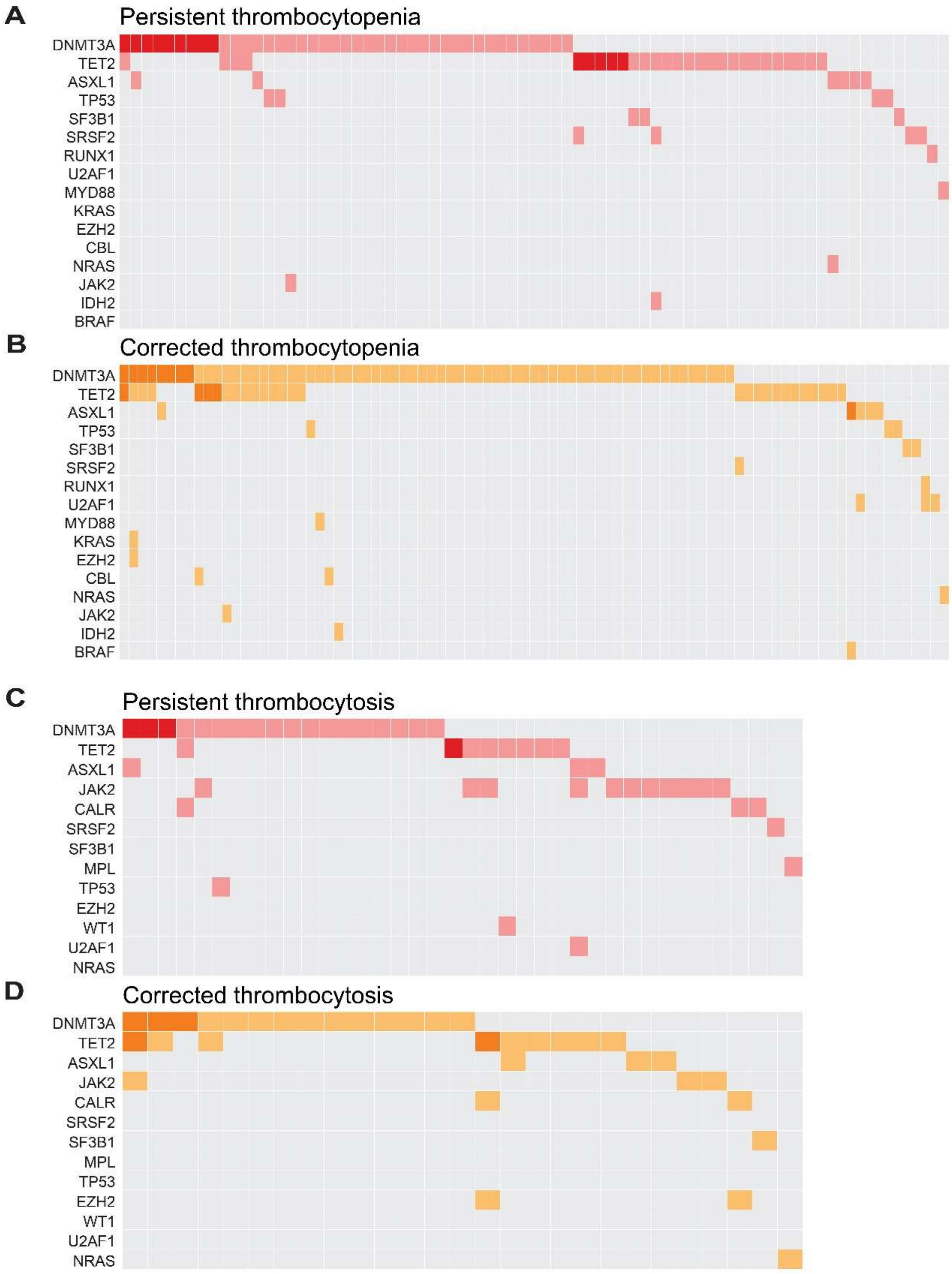

Supplemental Figure 16 – Overall survival stratified to the number of mutated genes and variant allele frequency in individuals with platelet count abnormalities

(A) Kaplan Meier graph showing OS for matched controls of thrombocytopenia cases, stratified by the number of mutated genes. (B) Kaplan Meier graph showing OS for matched controls of thrombocytopenia cases, stratified by maximum clone size. Kaplan Meier graph showing OS for thrombocytosis cases (C) and matched controls (D) according to the number of mutated genes. Kaplan Meier graph showing OS for individuals with thrombocytosis (E) and matched controls (F) according to clone size.

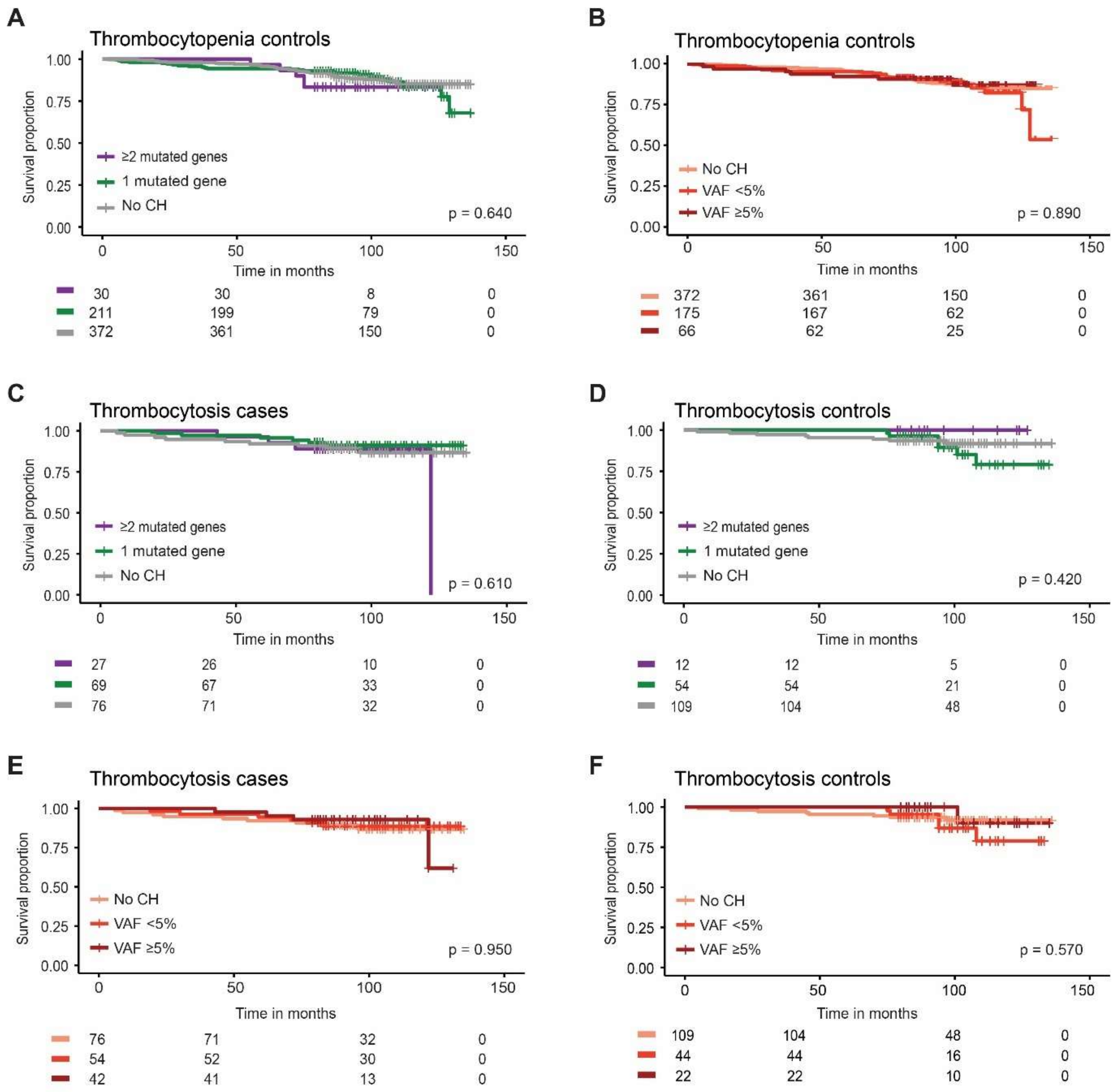

Supplement: Supplementary file 1 [file hs9-7-e821-s001.pdf]
